# Projected 21st-century changes in marine heterotrophic bacteria under climate change

**DOI:** 10.3389/fmicb.2023.1049579

**Published:** 2023-02-16

**Authors:** Heather H. Kim, Charlotte Laufkötter, Tomas Lovato, Scott C. Doney, Hugh W. Ducklow

**Affiliations:** ^1^Department of Marine Chemistry and Geochemistry, Woods Hole Oceanographic Institution, Woods Hole, MA, United States; ^2^Division of Climate and Environmental Physics, Physics Institute, University of Bern, Bern, Switzerland; ^3^Oeschger Centre for Climate Change Research, University of Bern, Bern, Switzerland; ^4^Ocean Modeling and Data Assimilation Division, Fondazione Centro Euro-Mediterraneo sui Cambiamenti Climatici - CMCC, Bologna, Italy; ^5^Department of Environmental Sciences, University of Virginia, Charlottesville, VA, United States; ^6^Department of Earth and Environmental Sciences, Columbia University, New York, NY, United States

**Keywords:** marine heterotrophic bacteria, bacteria, microbes, Coupled Model Intercomparison Project, CMCC-ESM, climate change

## Abstract

Marine heterotrophic *Bacteria* (or referred to as bacteria) play an important role in the ocean carbon cycle by utilizing, respiring, and remineralizing organic matter exported from the surface to deep ocean. Here, we investigate the responses of bacteria to climate change using a three-dimensional coupled ocean biogeochemical model with explicit bacterial dynamics as part of the Coupled Model Intercomparison Project Phase 6. First, we assess the credibility of the century-scale projections (2015–2099) of bacterial carbon stock and rates in the upper 100 m layer using skill scores and compilations of the measurements for the contemporary period (1988–2011). Second, we demonstrate that across different climate scenarios, the simulated bacterial biomass trends (2076–2099) are sensitive to the regional trends in temperature and organic carbon stocks. Bacterial carbon biomass declines by 5–10% globally, while it increases by 3–5% in the Southern Ocean where semi-labile dissolved organic carbon (DOC) stocks are relatively low and particle-attached bacteria dominate. While a full analysis of drivers underpinning the simulated changes in all bacterial stock and rates is not possible due to data constraints, we investigate the mechanisms of the changes in DOC uptake rates of free-living bacteria using the first-order Taylor decomposition. The results demonstrate that the increase in semi-labile DOC stocks drives the increase in DOC uptake rates in the Southern Ocean, while the increase in temperature drives the increase in DOC uptake rates in the northern high and low latitudes. Our study provides a systematic analysis of bacteria at global scale and a critical step toward a better understanding of how bacteria affect the functioning of the biological carbon pump and partitioning of organic carbon pools between surface and deep layers.

## 1. Introduction

Marine heterotrophic *Bacteria* (hereafter referred to as bacteria) respire a significant fraction of organic carbon produced in the surface ocean by photosynthesis into carbon dioxide (CO_2_) and remineralize organic matter into inorganic nutrients (Azam et al., 1983). Bacteria typically live as suspended cells on dissolved organic matter or, as attached cells on particulate organic matter, exhibiting functional diversity from oligotrophs (e.g., ubiquitous alphaproteobacterial clade SAR11; Morris et al., 2002) to copiotrophs (e.g., Flavobacteria; Baltar et al., 2015). In open oceans, bacteria account for the most of heterotrophic community respiration (Williams, 1981; Rivkin and Legendre, 2001) serving as a major sink for organic carbon and a source for CO_2_. A modest variation in the depth at which organic carbon is respired to CO_2_ by bacteria can drive changes in atmospheric CO_2_ concentrations of ~30 ppm (Kwon et al., 2009). Despite their importance in the ocean carbon cycle, the vast majority of three-dimensional (3-D) coupled physical-biogeochemical models do not simulate bacteria explicitly as a state variable and simply parameterize their impacts on particle flux attenuation (i.e., Martin curve).

Three types of controls have been hypothesized as the mechanistic drivers of bacterial dynamics, including temperature, substrate (organic carbon) availability, and grazing loss/viral mortality. In addition, bacterial activity is controlled by availability of nutrients (e.g., ammonium, phosphate, iron), but their effects are not typically as primary as availability of organic substrates (Eppley et al., 1977; Harrison, 1983; Pham et al., 2022). Any factors causing the changes in substrate availability and grazing/viral mortality are referred to as bottom-up and top-down control factors, respectively, while temperature increases metabolic rates directly (López-Urrutia and Morán, 2007; Kirchman et al., 2009; Morán et al., 2017) or acts as an interactive limiting factor with organic substrate for bacteria (Pomeroy and Wiebe, 2001; López-Urrutia and Morán, 2007; Huete-Stauffer et al., 2015). Early studies formulated the so-called Pomeroy Hypothesis that demonstrates the interplay between organic matter and temperature. According to this hypothesis, regardless of region, bacterial metabolism can be limited by substrate availability when temperature is near the annual minimum and, therefore, substrate availability can alleviate temperature limitation (Pomeroy and Deibel, 1986; Pomeroy et al., 1990, 1991; Wiebe et al., 1992). Global sea surface temperature has increased by 0.7°C for the last 100 years and is expected to increase over the next century (Bindoff et al., 2007) with consequences on the net primary production (NPP), inorganic nutrients, and organic substrates (Behrenfeld et al., 2001, 2006; Kwiatkowski et al., 2020). Studies suggest that bacteria are fairly resilient to ocean acidification (Grossart et al., 2006; Rochelle-Newall et al., 2011; Krause et al., 2012; Lindh et al., 2013; Roy et al., 2013; Oliver et al., 2014) but respond sensitively to changes in temperature and quality or quantity of organic substrates (Sarmento et al., 2010; Ducklow et al., 2011; Engel et al., 2014; Thornton, 2014) or inorganic nutrients (Cotner et al., 1997; Rivkin and Anderson, 1997; Caron et al., 2000). Warming has been shown to increase bacterial biomass, production, and respiration, decrease bacterial growth efficiency (BGE), and cause a taxonomic shift towards small-cells (Hoppe et al., 2008; Sarmento et al., 2010; Lara et al., 2013; Von Scheibner et al., 2014). However, the duration of these studies is limited and the mechanisms driving such changes remain inconclusive.

Here, we investigate the climate-driven responses in bacterial carbon stocks and rates for the next century (2015–2099) using the results of the CMCC Earth System Model (ESM) version 2 simulations (Lovato et al., 2022) produced under the Coupled Model Intercomparison Project Phase 6 (CMIP6). CMIP6 aims to better understand the past, present, and future long-term climate change arising from natural variability, or in response to the changes in radiative forcings defined by different Shared Socioeconomic Pathways (SSPs; Eyring et al., 2016). We first examine the variability and trends in bacterial stocks and rates under different SSPs both globally and regionally, and then discuss the underlying drivers of the projected responses. We take advantage of the first-order Taylor decomposition to isolate the effects of different factors controlling bacterial rates from one another to gain a better mechanistic understanding of the simulated responses.

## 2. Materials and methods

### 2.1. Model

Based on the Community Earth System Model, CMCC-ESM2 uses the Nucleus for European Modeling of the Ocean (NEMO v3.6; Madec & NEMO Team, 2016) for the ocean general circulation model and the global version of the Biogeochemical Flux Model (BFM 5.2; Vichi et al., 2020) for the biogeochemical component. The ocean model grid consists of 362 × 292 longitude/latitude and 50 vertical depth levels (i.e., 1–5,904 m, depth intervals varying from 1 m to 17 m in the upper 100 m and from 20 m to 400 m below) based on the ORCA tripolar grid at a nominal 1° latitude × longitude with a meridional refinement of 1/3° near the Equator. CMCC-ESM2 is run by CMCC (Fondazione Centro euro-Mediterraneo sui Cambiamenti Climatici, Italy) in native nominal resolutions of 100 km for both the ocean general circulation and biogeochemical models. The BFM equations solve the fluxes of C, N, P, Si, and Fe among living functional groups including bacteria, unicellular planktonic autotrophs (i.e., nano-phytoplankton, diatoms), zooplankton (i.e., micro- and mesozooplankton), and non-living functional groups for dissolved and particulate organic matter. In particular, phytoplankton groups and organic matter pools have a variable stoichiometry, while bacterial and zooplanktonic groups are modeled in terms of the sole carbon constituent with fixed stoichiometric ratios. The bacterial scheme in BFM simulates time-evolving dynamics of free-living and particle-attached bacteria explicitly. The BFM configuration used in CMCC-ESM2 is detailed in Lovato et al. (2022).

### 2.2. Simulations

For baseline simulations, we analyze the results of CMCC-ESM2 forced under CMIP6 historical simulations (1990–2013; Lovato et al., 2021a) for the reference patterns of bacterial stock and rates prior to warming as well as for model skill assessment. These historical simulations are forced by evolving, externally imposed solar variability, volcanic aerosols, and changes in atmospheric composition and land use caused by human activities to reflect observations (Eyring et al., 2016). For future simulations, we use the results of CMCC-ESM2 forced under 4 different SSPs of CMIP6 over the 21st century (2015–2100) including SSP1-2.6, SSP2-4.5, SSP3-7.0, and SSP5-8.5 (Lovato et al., 2021b,c,d,e). The SSPs describe the alternative evolutions of future society in the absence or with the varying degree of climate change and mitigation policy. SSP1-2.6 represents the lowest end of the future forcing pathways equivalent to the total radiative forcing level of 2.6 W m^−2^ in the year 2100. SSP2-4.5 represents the low/intermediate part of the future forcing pathways equivalent to 4.5 W m^−2^ in 2100. SSP3-7.0 represents the intermediate/high part of the future forcing pathways equivalent to 7.0 W m^−2^ in 2100. SSP5-8.5 represents the highest end of the future forcing pathways equivalent to 8.5 W m^−2^ in 2100 (O’Neill et al., 2016). These different SSPs are not all equally likely to occur but useful for differentiating the challenges for climate mitigation and adaptation. The future simulations relate to the baseline simulations where the end of the historical simulations (31 December 2014) serves as the start of each SSP projection (1 January 2015) with consistency ensured through the harmonization of emissions, concentrations, and land use across the simulations (O’Neill et al., 2016). We calculate the anomalies of each SSP-projected field relative to the climatology of the historical fields to facilitate comparisons among the simulations and different SSPs.

### 2.3. Variables

We analyze the depth-resolved, yearly mean fields of bacterial carbon biomass (mmol C m^−3^), bacterial organic carbon uptake rates (i.e., bacterial carbon demand, mmol C m^−3^ d^−1^, by definition), bacterial respiration rates (mmol C m^−3^ d^−1^), and potential water temperature (°C; Supplementary Table 1). Monthly mean fields of bacterial carbon biomass are additionally analyzed for model skill assessment. Other bacterial rate variables (e.g., the amount grazed by microzooplankton and lost due to mortality) have not been saved during the realization of CMIP6 experiments because the datasets analyzed here were produced specifically for CMIP6 experiments before the present study was undertaken (i.e., CMIP6 typically requests a limited set of the metrics and rates for marine biogeochemical variables including bacteria). We use the depth-averaged temperature in the upper 100 m layer (10–107 m), while the remaining variables are depth-integrated in the upper 100 m layer using the trapezoidal method. After depth averaging or integration, we do not further transform the model data given their approximate normal distributions with small skewness (tails) of the model-data residuals.

For model skill assessment, we utilize the bacterial measurement data from the global ocean compilation MARine Ecosystem biomass DATa (MAREDAT, *n* = 9,284 over 1° × 1° × 33 vertical layers × 12 months; Buitenhuis et al., 2012), Bermuda Atlantic Time-series Study (BATS; Steinberg et al., 2001), and Hawaii Ocean Time-series (HOT) at Station ALOHA (Lukas and Karl, 1999). MAREDAT is used for monthly comparisons between the model results and observations as it provides the monthly biomass data averaged from 1988 to 2011, while BATS and HOT data sets are used for both monthly and yearly comparisons. Model skill for bacterial biomass is evaluated against bacterial abundance (cell count) data. Due to general lack of bacterial carbon demand and respiration measurements, model skill for bacterial production (i.e., bacterial production = bacterial carbon demand—bacterial respiration) is evaluated against bacterial cellular carbon uptake rates measured *via* radioisotope incorporation methods. Bacterial abundance from BATS and HOT data sets is converted to bacterial carbon biomass using a conservative open-ocean carbon conversion factor of 10 fg C cell^−1^ (Ducklow and Carlson, 1992; Carlson and Ducklow, 1995). ^3^H-thymidine incorporation rate (pmol L^−1^ h^−1^) from BATS data sets is converted to bacterial production (mg C L^−1^ h^−1^) using a thymidine conversion factor of 3.0 × 10^18^ cells produced per mole of ^3^H-thymidine incorporated (Sanders et al., 2000). Bacterial production data are only available publicly at BATS.

### 2.4. Bacterial dynamics

The bacterial scheme in CMCC-ESM2 is derived from the previous BFM global ocean formulations (Vichi et al., 2007a,b) with a revised parameterization of bacterial growth dynamics (Lovato et al., 2022). The model represents a wide group of aerobic and anaerobic bacteria that utilize both particulate and dissolved organic matter as the source of growth. The time derivative of bacterial carbon biomass (mmol C m^−3^, Eq. 1) is driven by the sum of source and sink terms, as follows:


(1) $$\frac{\partial B}{\partial t}=BP–G–M,\;\mathrm{where}\;BP=BCD–R$$

where B is bacterial carbon biomass (Eqs. 2–4), BCD is bacterial carbon demand (Eqs. 5–7), R is bacterial respiration (Eq. 8), G is grazing (Eq. 9), M is mortality (Eq. 10), and BP is bacterial production (Eqs. (11) and (12)). Bacterial carbon demand is the sum of dissolved organic carbon (DOC) and particulate organic carbon (POC) uptake by bacteria. Mortality is a loss process dependent on temperature and biomass of bacteria, which is not related to grazing and viral lysis. Respiration, grazing, and mortality dynamics use the same formulations as in the previous BFM global ocean applications (BACT1; Vichi et al., 2020). The exception is NO_3_ consumption under anaerobic conditions that is computed using a constant C/N ratio (Paulmier et al., 2009) instead of the original reduction equivalent formulation. The source and sink terms available for analysis are bacterial carbon demand and respiration, while grazing and mortality have not been saved during CMIP6 experiments. There is no explicit nutrient limitation on bacterial growth. However, bacteria take up or release dissolved inorganic nutrients to maintain their internal nutrient ratios (C/N/P) depending on the nutrient content of the ingested substrates, which makes them behave as remineralizers or competitors to phytoplankton depending on their nutrient quota. The nutrient uptake rate of bacteria is regulated by the ambient nutrient concentrations, while the direct release of the nutrients occurs in the case of excess N or P with respect to the internal nutrient ratios (C/N/P) of bacteria (see below for details). This formulation provides the limitation on the nutrient uptake of bacteria indirectly in oligotrophic conditions.

As marine bacteria feed on both dissolved and particulate organic substrates, their carbon biomass (*B*, Eqs. 1 and 2) is accordingly broken down into free-living biomass (*B*_DOC_, Eq. 3) and particle-attached biomass (*B*_POC_, Eq. 4), given by:


(2) $$B=B_{\mathrm{DOC}}+B_{\mathrm{POC}}$$

(3) $$B_{\mathrm{DOC}}=B\times \varphi_{\mathrm{DOC}}$$

(4) $$B_{\mathrm{POC}}=B\times \left(1–\varphi_{\mathrm{DOC}}\right),\mathrm{where}\;\varphi_{\mathrm{DOC}}=\frac{DOC}{POC+DOC}$$

where *DOC* and *POC* represent total DOC and POC concentrations, respectively, and $\varphi_{\mathrm{DOC}}$ is the linear portion of the carbon content between the two substrate pools. The linear partition of bacterial biomass between the two substrate pools is a rather simple but effective assumption to explicitly account for feeding mechanisms distinct to free-living and particle-attached bacteria. Dissolved and particulate organic matter are simulated explicitly as prognostic state variables, including their respective content of N, P, and Fe. The total DOC and POC concentrations represent an ensemble of labile and semi-labile organic carbon materials produced and lost through the activities of the living functional groups (e.g., phytoplankton excretion, bacterial excretion and mortality, zooplankton mortality, and sloppy feeding by mesozooplankton). The release of labile organic substrates by phyto- and zooplankton accompanies the release of the nutrient contents of these substrates, while semi-labile pools are released only as carbon capsular material by phytoplankton without the release of the associated nutrients. Bacteria feed on DOC and POC as both are considered labile, but their capability to utilize each organic carbon pool depends on the nutrient quota of both bacteria and substrates. Bacteria are only allowed to take up organic matter to maintain their optimal nutrient quotas. If organic matter has high carbon content (i.e., when a relatively large portion of semi-labile substrates is released by phytoplankton), it makes the organic matter practically less labile because bacteria only can take up substrates to maintain their optimal N/C and P/C ratios. Thus, it is the interplay between the stoichiometric ratios of organic matter pools and bacteria that implicitly regulates the degree of lability of each substrate for bacterial utilization.

The carbon demands of both bacterial groups (mmol C m^−3^ d^−1^, Eq. 5) are given by:


(5) $$BCD=BCD_{\mathrm{DOC}}+BCD_{\mathrm{POC}}$$

where *BCD*, $BCD_{\mathrm{DOC}}$ , and $BCD_{\mathrm{POC}}$ represent the carbon demands of the whole bacterial community, free-living bacteria, and particle-attached bacteria, respectively.

The DOC uptake rate of free-living bacteria (mmol C m^−3^ d^−1^, Eq. 6) is parameterized using a multiplicative function of the maximum organic carbon uptake rate, temperature limitation factor, and DOC limitation factor, given by:

(6) $$\begin{array}{l} BCD_{\mathrm{DOC}}=\mu_{\mathrm{DOC}}\times B_{\mathrm{DOC}}\\ \;\mathrm{where}\;\mu_{\mathrm{DOC}}=\mu_{\max}\times Q_{10}^{B}\times \left(\frac{DOC^{3}}{DOC^{3}+X_{\mathrm{DOC}}^{3}}\right) \end{array}$$

where $\mu_{\max}$ is the maximum specific organic carbon uptake rate, $Q_{10}^{B}$ parameterizes the temperature limitation factor, and $X_{\mathrm{DOC}}$ is the half-saturation coefficient of DOC. Details on the parameter choice, values, and references are available in Supplementary Table 3. The temperature limitation factor simulates the impact of temperature on bacterial substrate uptake rates (i.e., temperature control). A Monod-like equation is employed to simulate the interaction of free-living bacteria with DOC, which reflects that DOC concentration is the only limiting factor for free-living bacterial uptake. A cubic sigmoid is adopted to simulate an enhanced bacterial response (i.e., an arbitrary choice to simulate a faster response under favorable growth conditions) under optimal conditions and its strong reduction when substrate limitation factors become more relevant.

The POC uptake rate of particle-attached bacteria (mmol C m^−3^ d^−1^, Eq. 7) is parameterized using a multiplicative function of the maximum organic carbon uptake rate, depth limitation factor, POC limitation factor, and particle-attached biomass, given by:


(7) $$\begin{array}{l} BCD_{\mathrm{POC}}=\mu_{\mathrm{POC}}\times B_{\mathrm{POC}}\\ \;\mathrm{where}\;\mu_{\mathrm{POC}}=\mu_{\max}\times \min\left(1,{\left(\frac{z}{z_{0}}\right)}^{b}\right)\times \left(\frac{POC^{3}}{POC^{3}+{\left(X_{\mathrm{POC}}\times B_{\mathrm{POC}}\right)}^{3}}\right)\; \end{array}$$

where min (1, ${\left(\frac{z}{z_{0}}\right)}^{b}$ ) parameterizes the depth limitation factor and $X_{\mathrm{POC}}$ is the half-saturation coefficient of POC. The decline in particle-attached biomass, as highlighted by recent observations (Grossart and Gust, 2009; Thiele et al., 2015), is simulated through a depth limitation factor similar to the Martin Curve (Martin et al., 1987). The Contois formulation is used for particle-attached colonies (Wang and Li, 2014) to reflect that particle-attached bacteria tend to form attached colonies that primarily grow as a function of POC concentration and biomass of particle-attached bacteria. As in Eq. 6, a cubic function is adopted to represent a fast bacterial response to substrates availability.

Bacterial respiration represents nutrient remineralization (i.e., organic matter respiration) and chemotrophic activities (e.g., denitrification), which change as a function of temperature, bacterial biomass, and dissolved oxygen concentration. An additional respiration cost is included to simulate lower metabolic efficiency under anaerobic conditions, which is regulated through a steep sigmoidal function of dissolved oxygen concentration (Vichi et al., 2004). Bacterial respiration (mmol C m^−3^ d^−1^, Eq. 8) is given by:


(8) $$R=b_{B}\times Q_{10}^{B}\times B+\left[\gamma_{B}^{a}+\gamma_{B}^{o}\times f_{B}^{o}\right]\times BCD$$

where $b_{B}$ is the respiration rate, $\gamma_{B}^{a}$ is the fraction of active respiration, $\gamma_{B}^{o}$ is the fraction of additional respiration under low oxygen concentrations, and $f_{B}^{o}$ is the nondimensional oxygen regulating factor. The oxygen regulating factor is defined as $f_{B}^{o}=\frac{{\left(DO\right)}^{3}}{{\left(DO\right)}^{3}+{\left(h_{B}^{o}\right)}^{3}}$ where DO is dissolved oxygen concentration and $h_{B}^{o}$ is the half-saturation term.

Grazing (mmol C m^−3^ d^−1^, Eq. 9) is parameterized using a Type 2 formulation (Gentleman et al., 2003), given by:


(9) $$G=r_{Z}^{0}\times Q_{10}^{Z}\times \frac{\delta_{Z,B}\;e_{Z,B}\;B}{F_{c}}\times \frac{F_{c}}{F_{c}+h_{Z}^{F}}\times Z$$

where *Z* is microzooplankton biomass, $r_{Z}^{ο}$ is the potential specific growth rate of microzooplankton, $\delta_{Z,B}$ is the feeding affinity on bacteria, $h_{Z}^{F}$ is the total food ingestion potential, $e_{Z,B}\;$ is the capture efficiency of bacteria, and $F_{c}$ is the potential food availability. The potential food availability is defined as $F_{c}=\underset{X=B,P}{\sum }\delta_{Z,X}\times e_{Z,X}\times X_{s}$ where *X* is the biomass of bacteria (*B*) and phytoplankton (*P*). The capture efficiency is defined as $e_{Z,X}$ = $\frac{X}{X+\mu_{Z}}$ where $\mu_{Z}$ is the feeding threshold. In Equation (9), we show *F_c_* twice to emphasize the third and fourth terms, which represent uptake of the given prey to total uptake of all preys and limiting function on total uptake, respectively.

Mortality (mmol C m^−3^ d^−1^, Eq. 10) is given by:


(10) $$M=d_{B}\times Q_{10}^{B}\times B$$

where $d_{B}$ is the mortality rate.

### 2.5. Model skill scores

The credibility of the SSPs projections is assessed by evaluating present-day model performance for the historical baseline period (1990–2013). We base model skill assessment on the goodness-of-fit of the model results versus the observational data, as well as on different univariate skill scores including average bias, average absolute error, root mean square difference (RMSD), correlation coefficients, and reliability index (Leggett and Williams, 1981). The average bias and average absolute error measure the distance between a set of observations and model predictions. RMSD quantifies the scale of an offset between the model results and observational data for any given point and relates to the average bias and centered (unbiased) RMSD (i.e., RMSD^2^ = average bias^2^ + centered RMSD^2^). The reliability index is interpreted as a value such that 68% of model predictions fall within the value of the reliability index and its reciprocal (Smith and Rose, 1995). We limit model skill assessment to bulk bacterial biomass data given the lack of free-living- or particle-attached- bacterial biomass. Model skill for other variables (e.g., particle export fluxes for POC) is demonstrated in Lovato et al. (2022).

## 3. Results

### 3.1. Model skill assessment

Globally, the model captures the monthly biomass averages with ~35% differences between the model results and observations. The model captures the monthly biomass averages with ~15% and ~7% differences for BATS and HOT, respectively, while the yearly biomass averages are better captured at both sites with <~3% differences between the model results and observations (Supplementary Table 2). There are similar variances (i.e., standard deviation, Supplementary Table 2) between the observations and model results globally (Supplementary Figure 1) and at BATS for the monthly averages (Supplementary Figure 3) and at HOT for the yearly averages (Supplementary Figure 2), while the standard deviation of the model results is smaller than that of the observations at BATS for the yearly averages (Supplementary Figure 3). The model biases (i.e., model results – observations) tend to increase with the model biomass values (Supplementary Figures 1–3). The model well captures the monthly biomass variability both globally (*r* = 0.24, *p* = 0.02) and at BATS (*r* = 0.38, *p* = 0.005; Supplementary Table 2). Generally, there are slight to moderate, negative model biases for the biomass averages. RMSD and centered RMSD are relatively high for the global comparisons and low for the regional comparisons. The reliability indices indicate that 68% of the monthly biomass averages from the model results fall within 56–178%, 65–145%, and 77–129% of those from the observations for globally, BATS, and HOT, respectively, while 68% of the yearly biomass averages from the model fall within 86–116% and 82–123% of those from the observations for BATS and HOT, respectively. Bacterial production shows relatively poor model skill because of model overestimation of the yearly averages by ~4 times at BATS, while other metrics are similar as those assessed for biomass (Supplementary Table 2, Supplementary Figure 4).

### 3.2. Global trends

**Figure 1:**
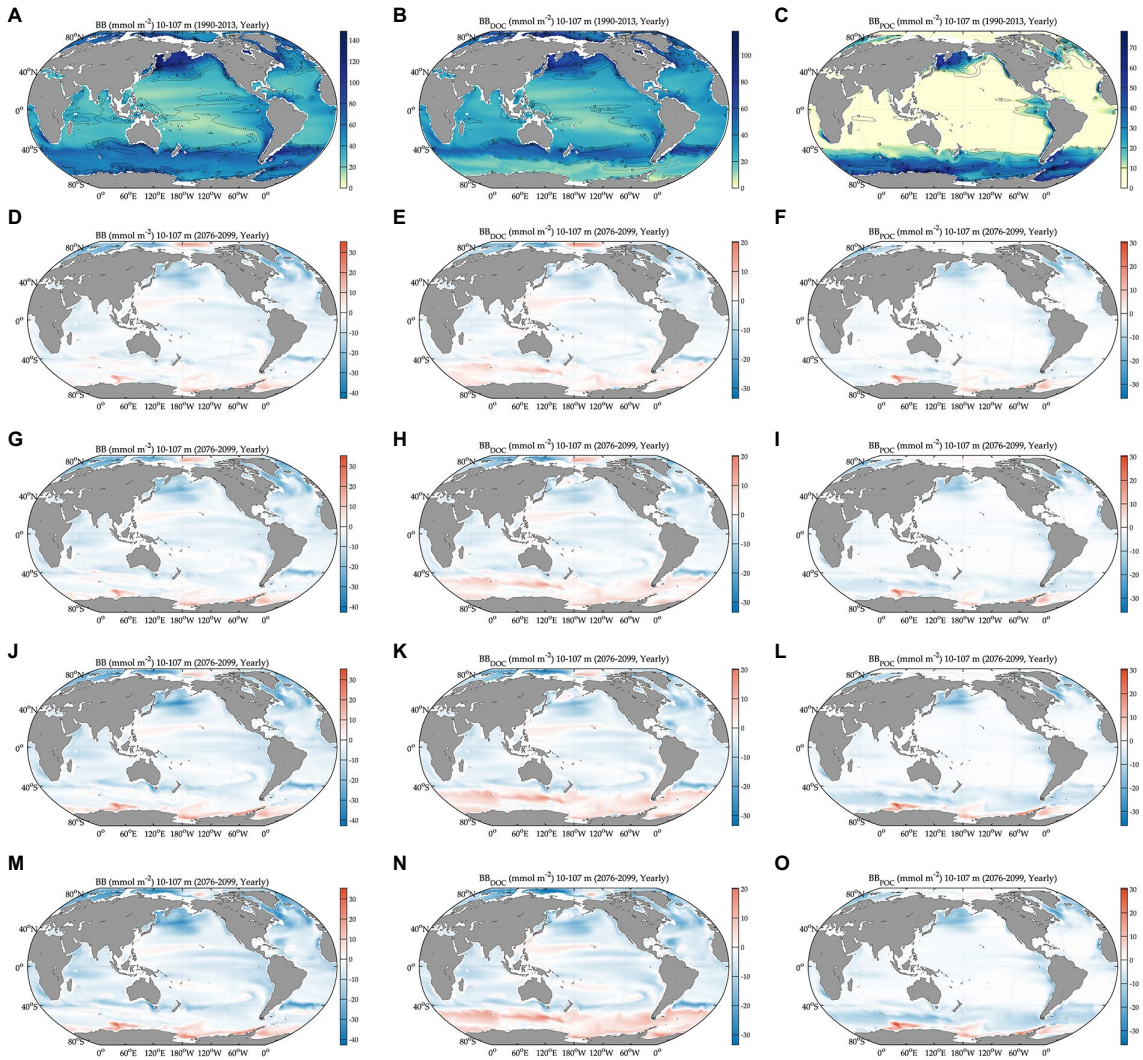
Global projections of bacterial carbon biomass during the baseline period (1990–2013) and their anomalies under different climate change scenarios relative to the baseline period (2076–2099). BB: bacterial carbon biomass, BB_DOC_: biomass of free-living bacteria, and BB_POC_: biomass of particle-attached bacteria. Bacterial carbon biomass during the baseline period **(A)** and its change relative to **(A)** under different SSPs **(D,G,J,M)**. Carbon biomass of free-living bacteria during the baseline period **(B)** and its change relative to **(B)** under different SSPs **(E,H,K,N)**. Carbon biomass of particle-attached bacteria during the baseline period **(C)** and its change relative to **(C)** under different SSPs **(F,I,L,O)**. All variables are depth-integrated in the upper 100 m. Solid-line contours as standard deviation from averaging over 1990–2013. Anomalies are 2076–2099 average values relative to 1990–2013 average values. SSP1–2.6: total radiative forcing of 2.6 W m^−2^ in the year 2100, SSP2–4.5: total radiative forcing of 4.5 W m^−2^ in 2100, SSP3–7.0: total radiative forcing of 7.0 W m^−2^ in 2100, and SSP5–8.5: total radiative forcing of 8.5 W m^−2^ in 2100. The figures are in the order of increasing SSPs from top to bottom.

**Figure 2:**
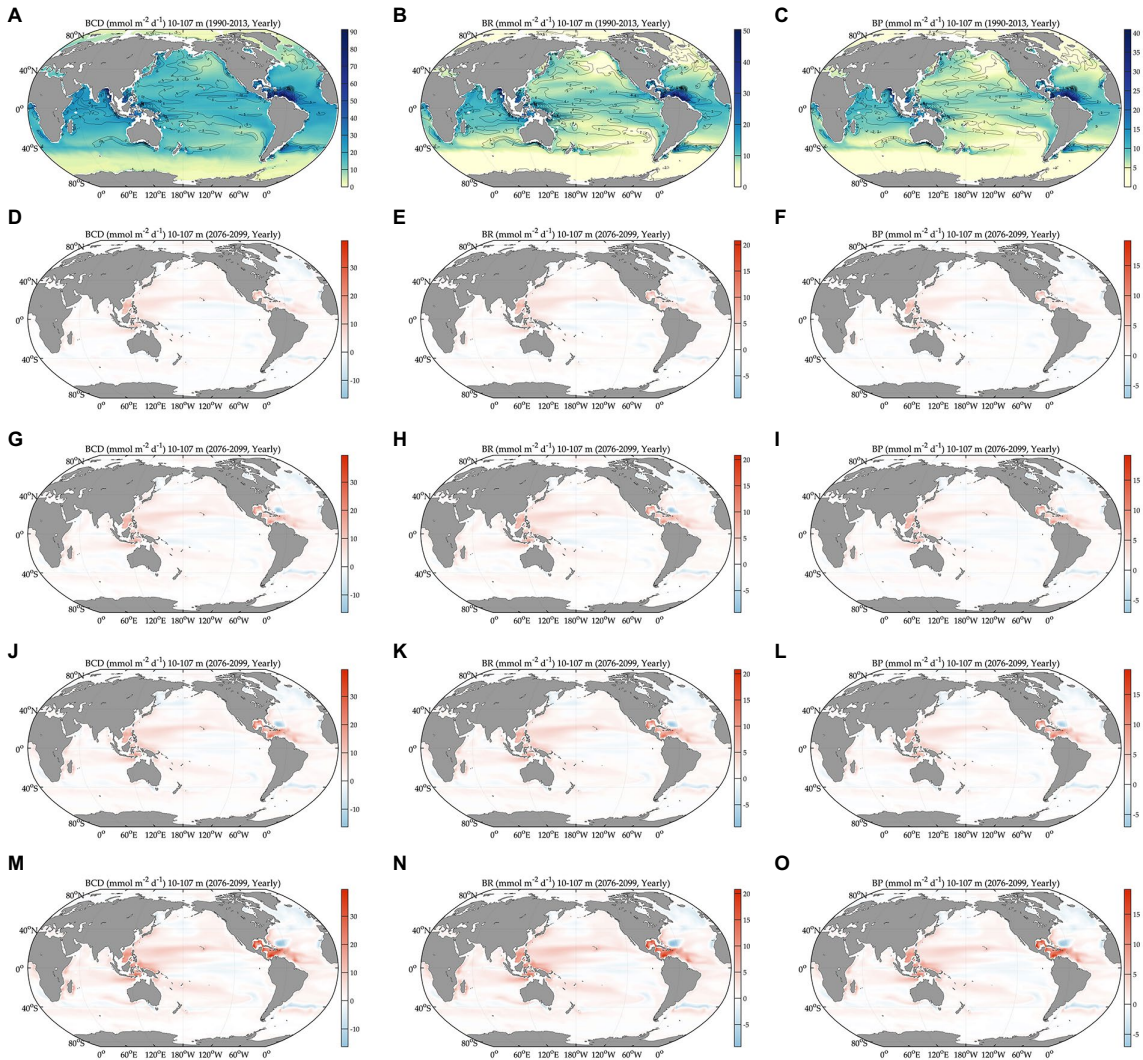
Global projections of bacterial carbon rates during the baseline period (1990–2013) and their anomalies under different climate change scenarios relative to the baseline period (2076–2099). BCD: bacterial carbon demand, BR: bacterial respiration, and BP: bacterial production. Bacterial carbon demand during the baseline period **(A)** and its change relative to **(A)** under different SSPs **(D,G,J,M)**. Bacterial respiration during the baseline period **(B)** and its change relative to **(B)** under different SSPs **(E,H,K,N)**. Bacterial production during the baseline period **(C)** and its change relative to **(C)** under different SSPs **(F,I,L,O)**. The figures are in the order of increasing SSPs from top to bottom.

**Figure 3:**
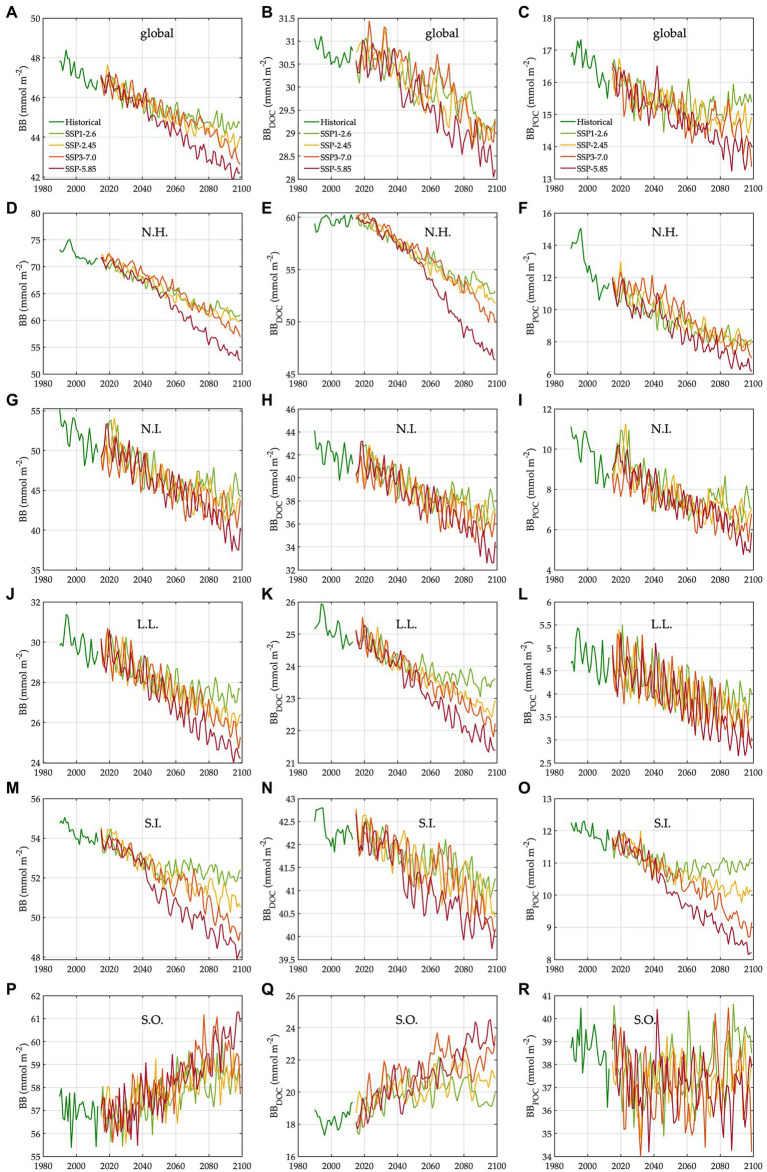
Trends in bacterial carbon biomass under different climate change scenarios. N.H.: northern high latitudes, N.I.: northern intermediates, L.L.: low latitudes, S.I.: southern intermediates, and S.O.: Southern Ocean. The trends (1990–2099) in bacterial carbon biomass in the global ocean **(A)**, N.H. **(D)**, N.I. **(G)**, L.L. **(J)**, S.I. **(M)**, and S.O. **(P)**. The trends (1990–2099) in carbon biomass of free-living bacteria in the global ocean **(B)**, N.H. **(E)**, N.I. **(H)**, L.L. **(K)**, S.I. **(N)**, and S.O. **(Q)**. The trends (1990–2099) in carbon biomass of particle-attached bacteria in the global ocean **(C)**, N.H. **(F)**, N.I. **(I)**, L.L. **(L)**, S.I. **(O)**, and S.O. **(R)**.

**Figure 4:**
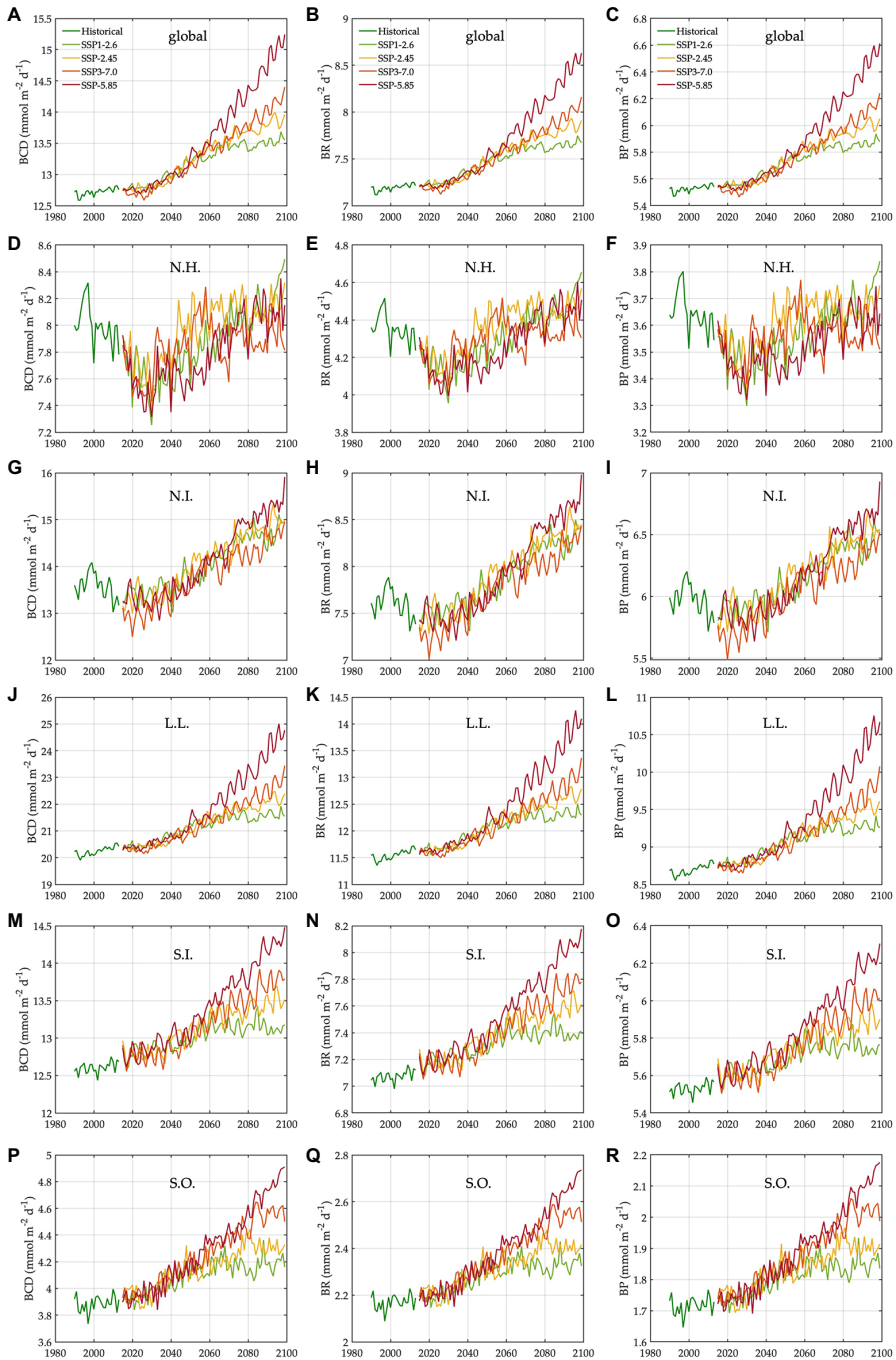
Trends in bacterial carbon rates under different climate change scenarios. N.H.: northern high latitudes, N.I.: northern intermediates, L.L.: low latitudes, S.I.: southern intermediates, and S.O.: Southern Ocean. The trends (1990–2099) in bacterial carbon demand in the global ocean **(A)**, N.H. **(D)**, N.I. **(G)**, L.L. **(J)**, S.I. **(M)**, and S.O. **(P)**. The trends (1990–2099) in bacterial respiration in the global ocean **(B)**, N.H. **(E)**, N.I. **(H)**, L.L. **(K)**, S.I. **(N)**, and S.O. **(Q)**. The trends (1990–2099) in bacterial production in the global ocean **(C)**, N.H. **(F)**, N.I. **(I)**, L.L. **(L)**, S.I. **(O)**, and S.O. **(R)**.

During the baseline period, the global averages of total bacterial carbon biomass, bacterial carbon demand, free-living biomass, particle-attached biomass, bacterial respiration, and bacterial production are 47 ± 0.07 mmol C m^−2^, 13 ± 0.03 mmol C m^−2^ d^−1^, 31 ± 0.05 mmol C m^−2^, 17 ± 0.05 mmol C m^−2^, 7.2 ± 0.02 mmol C m^−2^ d^−1^, and 5.5 ± 0.01 mmol C m^−2^ d^−1^, respectively (Figures 1, 2, Table 1). The warming-driven changes in bacterial carbon stocks and rates and other variables compared to those for the baseline period are the largest under SSP5-8.5, followed by SSP3-7.0, SSP2-4.5, and SSP1-2.6 (Figures 1–4, Supplementary Figures 5–9). The global mean temperature in the upper 100 m layer increases by 1.2 ± 0.05°C to 2.5 ± 0.05°C (i.e., calculated as the 2076–2099 average minus the 1990–2013 average; Table 1, Supplementary Figures 8, 9). Under warming, the global mean bacterial biomass decreases by 5–10% (i.e., calculated as the 2076–2099 average divided by the 1990–2013 average; Table 1). Free-living and particle-attached bacteria constitute 65% and 35% of the whole bacterial community prior to warming, and warming increases the free-living portion by 1–2%. Warming causes twice larger biomass declines for particle-attached bacteria (7–15%) than their free-living counterparts (4–6%; Table 1). In contrast to the biomass responses, warming increases bacterial carbon demand, respiration, and production similarly, by 6–15% (Table 1).

**Table 1:** Composites for baseline and different climate change scenarios.

Region	Global ocean	N.H. (>50°N)	N.I. (30°N–50°N)	L.L. (30°S–30°N)	S.I. (30°S-50°S)	S.O. (>50°S)
Area in %		11	8	39	14	28
*n*	61,425	6,729	4,814	24,070	8,566	17,246
Mean BB ± SE (mmol C m^−2^)						
Baseline	47 ± 0.07	72 ± 0.15	52 ± 0.23	30 ± 0.06	54 ± 0.20	57 ± 0.07
SSP1–2.6	45 ± 0.06	62 ± 0.14	45 ± 0.20	27 ± 0.05	52 ± 0.19	58 ± 0.06
SSP2–4.5	44 ± 0.06	61 ± 0.14	44 ± 0.20	26 ± 0.05	51 ± 0.19	59 ± 0.07
SSP3–7.0	44 ± 0.06	60 ± 0.14	42 ± 0.19	26 ± 0.05	50 ± 0.19	59 ± 0.07
SSP5–8.5	43 ± 0.06	55 ± 0.14	41 ± 0.19	25 ± 0.05	49 ± 0.19	60 ± 0.07
Mean BB_DOC_ ± SE (mmol C m^−2^)						
Baseline	31 ± 0.05	60 ± 0.11	42 ± 0.13	25 ± 0.04	42 ± 0.13	18 ± 0.05
SSP1–2.6	29 ± 0.05	54 ± 0.10	38 ± 0.12	24 ± 0.04	41 ± 0.12	20 ± 0.05
SSP2–4.5	29 ± 0.05	53 ± 0.10	37 ± 0.12	23 ± 0.04	41 ± 0.12	21 ± 0.05
SSP3–7.0	29 ± 0.04	52 ± 0.10	36 ± 0.12	23 ± 0.04	41 ± 0.12	22 ± 0.05
SSP5–8.5	29 ± 0.04	48 ± 0.10	35 ± 0.11	22 ± 0.04	40 ± 0.12	23 ± 0.05
Mean BB_POC_ ± SE (mmol C m^−2^)						
Baseline	17 ± 0.05	13 ± 0.08	9.7 ± 0.11	4.8 ± 0.03	12 ± 0.11	39 ± 0.07
SSP1–2.6	15 ± 0.05	8.2 ± 0.07	7.2 ± 0.09	3.9 ± 0.02	11 ± 0.10	39 ± 0.07
SSP2–4.5	15 ± 0.05	8.2 ± 0.07	7.0 ± 0.09	3.6 ± 0.02	10 ± 0.09	38 ± 0.08
SSP3–7.0	15 ± 0.05	7.9 ± 0.07	6.3 ± 0.08	3.5 ± 0.02	9.4 ± 0.09	38 ± 0.08
SSP5–8.5	14 ± 0.05	7.0 ± 0.06	6.0 ± 0.08	3.2 ± 0.02	8.7 ± 0.08	37 ± 0.08
Mean BCD ± SE (mmol C m^−2^ d^−1^)						
Baseline	13 ± 0.03	8.0 ± 0.04	14 ± 0.04	20 ± 0.04	13 ± 0.06	3.9 ± 0.01
SSP1–2.6	14 ± 0.03	8.2 ± 0.04	15 ± 0.04	22 ± 0.05	13 ± 0.05	4.2 ± 0.01
SSP2–4.5	14 ± 0.03	8.1 ± 0.04	15 ± 0.04	22 ± 0.06	13 ± 0.05	4.3 ± 0.01
SSP3–7.0	14 ± 0.03	7.9 ± 0.04	14 ± 0.04	22 ± 0.06	14 ± 0.06	4.5 ± 0.01
SSP5–8.5	15 ± 0.04	8.1 ± 0.04	15 ± 0.05	24 ± 0.07	14 ± 0.06	4.7 ± 0.01
Mean BR ± SE (mmol C m^−2^ d^−1^)						
Baseline	7.2 ± 0.02	4.4 ± 0.02	7.6 ± 0.02	12 ± 0.02	7.1 ± 0.03	2.2 ± 0.01
SSP1–2.6	7.7 ± 0.02	4.5 ± 0.02	8.3 ± 0.02	12 ± 0.03	7.4 ± 0.03	2.3 ± 0.01
SSP2–4.5	7.8 ± 0.02	4.5 ± 0.02	8.4 ± 0.02	13 ± 0.03	7.6 ± 0.03	2.4 ± 0.01
SSP3–7.0	7.9 ± 0.02	4.3 ± 0.02	8.2 ± 0.02	13 ± 0.03	7.7 ± 0.03	2.5 ± 0.01
SSP5–8.5	8.3 ± 0.02	4.5 ± 0.02	8.6 ± 0.03	14 ± 0.04	8.0 ± 0.03	2.6 ± 0.01
Mean BP ± SE (mmol C m^−2^ d^−1^)						
Baseline	5.5 ± 0.01	3.7 ± 0.02	6.0 ± 0.02	8.7 ± 0.02	5.5 ± 0.03	1.7 ± 0.01
SSP1–2.6	5.9 ± 0.01	3.7 ± 0.02	6.4 ± 0.02	9.3 ± 0.02	5.8 ± 0.02	1.8 ± 0.01
SSP2–4.5	6.0 ± 0.01	3.7 ± 0.02	6.5 ± 0.02	9.5 ± 0.02	5.9 ± 0.02	1.9 ± 0.01
SSP3–7.0	6.1 ± 0.01	3.6 ± 0.02	6.3 ± 0.02	9.6 ± 0.03	6.0 ± 0.03	2.0 ± 0.01
SSP5–8.5	6.4 ± 0.02	3.6 ± 0.02	6.6 ± 0.02	10 ± 0.03	6.1 ± 0.03	2.1 ± 0.01
Mean temperature ± SE (°C)						
Baseline	13 ± 0.03	2.2 ± 0.03	14 ± 0.05	24 ± 0.02	13 ± 0.04	0.83 ± 0.02
SSP1–2.6	14 ± 0.03	4.7 ± 0.03	17 ± 0.04	25 ± 0.02	14 ± 0.04	1.2 ± 0.02
SSP2–4.5	14 ± 0.03	5.0 ± 0.03	17 ± 0.04	26 ± 0.02	15 ± 0.04	1.5 ± 0.02
SSP3–7.0	15 ± 0.03	5.1 ± 0.02	17 ± 0.04	26 ± 0.02	15 ± 0.04	1.7 ± 0.02
SSP5–8.5	15 ± 0.03	6.4 ± 0.02	18 ± 0.04	27 ± 0.02	16 ± 0.04	2.0 ± 0.02
Mean POC ± SE (mmol C m^−2^)						
Baseline	420 ± 0.76	369 ± 2.1	365 ± 2.7	323 ± 0.9	394 ± 2.6	602 ± 1.0
SSP1–2.6	399 ± 0.76	284 ± 1.9	306 ± 2.5	299 ± 0.9	383 ± 2.5	616 ± 1.0
SSP2–4.5	394 ± 0.75	284 ± 1.9	302 ± 2.4	294 ± 0.9	370 ± 2.4	614 ± 1.0
SSP3–7.0	388 ± 0.76	274 ± 1.8	281 ± 2.2	287 ± 0.9	353 ± 2.4	621 ± 1.1
SSP5–8.5	377 ± 0.77	255 ± 1.8	273 ± 2.3	274 ± 0.9	339 ± 2.3	618 ± 1.1
Mean DOC ± SE (mmol C m^−2^)						
Baseline	1703 ± 3.0	2,174 ± 3.9	2,269 ± 2.9	2,350 ± 2.1	1984 ± 6.3	318 ± 1.4
SSP1–2.6	1772 ± 3.0	2,330 ± 2.9	2,386 ± 2.7	2,416 ± 2.1	2032 ± 5.9	354 ± 1.5
SSP2–4.5	1790 ± 3.0	2,321 ± 2.8	2,384 ± 2.7	2,432 ± 2.1	2070 ± 5.6	383 ± 1.6
SSP3–7.0	1811 ± 3.0	2,318 ± 2.8	2,400 ± 2.7	2,442 ± 2.1	2,115 ± 5.3	415 ± 1.7
SSP5–8.5	1846 ± 3.0	2,332 ± 2.5	2,433 ± 2.7	2,481 ± 2.1	2,160 ± 5.1	451 ± 1.8

BB, bacterial carbon biomass; BCD, bacterial carbon demand; BB_DOC_, biomass of free-living bacteria; BB_POC_, biomass of particle-attached bacteria; BR, bacterial respiration; BP, bacterial production; POC, particulate organic carbon; and DOC, dissolved organic carbon. Water temperature is averaged in the upper 100 m layer; while the rest variables are depth-integrated in the upper 100 m layer. SE, standard error; *n*, sample size. N.H., northern high latitudes; N.I., northern intermediates; L.L., low latitudes; S.I., southern intermediates; and S.O., Southern Ocean.

### 3.3. Regional trends and variability

The regional mean temperature in the upper 100 m layer rises by 2.5–4.2°C in the northern high latitudes (i.e., >50°N), 2.3°C to 3.6°C in the northern intermediates (i.e., 30–50°N), 1.3–2.7°C in the low latitudes (i.e., 30°S–30°N), 1.0–2.5°C in the southern intermediates (i.e., 30–50°S), and 0.38°C to 1.2°C in the Southern Ocean (i.e., >50°S; Table 1, Supplementary Figure 9). Warming is associated with the declines in total bacterial biomass in most regions, with the largest relative changes in the northern high latitudes (14–24%) followed by the northern intermediates (13–21%), low latitudes (8–16%), and southern intermediates (4–10%; Table 1). By contrast, an opposite trend occurs in the Southern Ocean in which warming drives the biomass increases by 3–5%, reversing the initial biomass difference between the two high-latitude oceans for the baseline period (Table 1). The low latitudes are characterized by the lowest biomass averages among all regions, regardless of warming.

The Southern Ocean is distinct in the way that a major fraction (61–68%) of the whole bacterial community constitutes particle-attached cells because of more POC than DOC (Eqs. 3 and 4), in opposition to other regions where only 12–22% of the total biomass are particle-attached biomass (Table 1). In the northern high latitudes, warming results in the largest biomass declines for both free-living (10–19%) and particle-attached bacteria (35–45%), followed by the northern intermediates (10–17% vs. 26–38%), low latitudes (6–13% vs. 19–34%), and southern intermediates (3–5% vs. 8–27%). In the Southern Ocean, particle-attached biomass decreases only slightly (0.3–5%) while free-living biomass increases greatly (8–27%), driving the net biomass increases in response to warming. Under warming, all bacterial rate variables increase in most regions, except the northern high latitudes where bacterial production decreases slightly under SSP5-8.5 and SSP3-7.0 because of the larger warming-driven increases in bacterial respiration than those in bacterial carbon demand. Bacterial carbon demand and respiration change similarly within the region, driving the increases in bacterial production for most regions except for the northern high latitudes. The percent increases in bacterial rates under warming are larger in the Southern Ocean (8–21%; Table 1) than other regions or globally (6–15%; Table 1).

### 3.4. Underlying mechanisms

The changes in bacterial biomass are driven by the changes in the source term (i.e., bacterial production; *B = BCD – R* in Eq. 1) and sink terms (i.e., grazing, mortality; *G, M* in Eq. 1), control of which are referred to as bottom-up and top-down controls, respectively. In theory, one can decompose the changes in bacterial biomass into those driven by the changes in bottom-up and top-down controls, respectively, but none of the sink terms were saved during CMIP6 experiments. At best, we can only compare bottom-up and top-down controls qualitatively based on the simulated biomass trends (e.g., the net biomass decline is driven by an overall larger biomass loss *via* top-down control than biomass production *via* bottom-up control). The net rate of change of bacterial biomass (*dB/dt*) is typically small relative to the source (*BP*) and sink (*G, M*) terms, and on the bacterial population time-scales of a few days the population dynamics can be approximated as in steady state where the source is balanced by sinks (i.e., *BP* ~ =*G* + *M*; Eq. 1). Based on available model diagnostics, mortality is likely a larger loss term than grazing both globally and regionally (i.e., mortality accounts for 58–60% and 43–79% of bacterial production normalized by bacterial biomass globally and regionally). The relative proportion of mortality versus grazing sinks will evolve under climate change but the expectation, based again on existing model output, is that mortality will remain the dominant bacterial sink term in a warmer future ocean.

The changes in bacterial production are driven by the changes in bacterial carbon demand and respiration (Eq. 1). The results from the global and regional composites (Sections 3.2, 3.3) demonstrate that BGE (i.e., calculated as the ratio of bacterial production to bacterial carbon demand) is highly consistent across different regions and SSPs for the given region (~0.44; Table 1), with only slight changes over seasons and depth levels. Therefore, bacterial carbon demand is a main determinant of bacterial production despite other regulating factors such as dissolved oxygen concentration. This allows us to simplify the changes in bacterial carbon demand into the changes in bacterial production, as follows:


(11) $$\begin{array}{l} BP_{\mathrm{DOC}}\sim 0.44\times \mu_{\mathrm{DOC}}\times B_{\mathrm{DOC}}\;\;\mathrm{where}\;\mu_{\mathrm{DOC}}=\\ \;\;\;\;\;\;\;\;\;\;\;\;\;\;\;\;\;\;\;\;\;\;\;\;\mu_{\max}\times Q_{10}^{\frac{T-10}{10}}\times \left(\frac{DOC^{3}}{DOC^{3}+X_{\mathrm{DOC}}^{3}}\right)\; \end{array}$$

(12) $$\begin{array}{l} BP_{\mathrm{POC}}\sim 0.44\times \mu_{\mathrm{POC}}\times B_{\mathrm{POC}}\;\;\mathrm{where}\;\mu_{\mathrm{POC}}=\\ \mu_{\max}\times \min\;\left(1,{\left(\frac{z}{z_{0}}\right)}^{b}\right)\times \left(\frac{POC^{3}}{POC^{3}+{\left(X_{\mathrm{POC}}\times B_{\mathrm{POC}}\right)}^{3}}\right)\; \end{array}$$

where *BP*_DOC_ and *BP*_POC_ is bacterial production specific to free-living and particle-attached cells, respectively. Temperature and DOC concentration determine DOC uptake rates of free-living cells (Eq. 6). Temperature, POC concentration, and bacterial biomass determine POC uptake rates of particle-attached cells (Eq. 7) wherein temperature effect is embedded *via* depth-dependent attenuation of the biomass.

We can assess a main driver of the changes in DOC uptake rates of free-living bacteria (Eq. 6) by conducting the first-order Taylor decomposition (as in Laufkötter et al., 2016) that decomposes the given changes in DOC uptake rates to those driven by the changes in the temperature limitation factor and the DOC limitation factor, as follows:


(13) $$\begin{array}{l} \frac{\partial \mu_{\mathrm{DOC}}}{\partial t}=\mu_{\max}\times \frac{\partial }{\partial t}\left(\frac{DOC^{3}}{DOC^{3}+X_{\mathrm{DOC}}^{3}}\right)\times Q_{10}^{\frac{T-10}{10}}\\ +\mu_{\max}\times \frac{\partial }{\partial t}\left(Q_{10}^{\frac{T-10}{10}}\right)\times \left(\frac{DOC^{3}}{DOC^{3}+X_{\mathrm{DOC}}^{3}}\right)+\mathrm{residual}\; \end{array}$$

where the first term on the right-hand side represents the changes in DOC uptake rates driven by the changes in DOC control (i.e., changes in DOC control weighted with temperature control), while the second term represents the changes in DOC uptake rates driven by the changes in temperature control (i.e., changes in temperature control weighted with DOC control). The difference between the 1990–2013 average and the 2076–2099 average is used as an estimate for the partial derivatives, while the 1990–2013 average is used as an estimate for weighting variables. The Taylor decomposition into temperature and substrate control is only possible for free-living bacteria (Eq. 6) because the coupling of particle-attached biomass and POC uptake rates (as in the denominator in Eq. 7) makes it difficult to decompose the changes in POC uptake rates to those driven by the changes in temperature and POC controls. The same argument holds for the whole community since it contains particle-attached cells.

The results of the Taylor decomposition demonstrate that the residuals are small (i.e., 1–13% the true changes in DOC uptake rates), allowing us to assess the impacts of temperature and DOC controls on the given changes in DOC uptake rates of free-living bacteria. DOC and temperature controls act in the same direction, reflecting the positive effects of increases in temperature and DOC concentrations on DOC uptake rates of free-living bacteria. Globally, the changes in DOC uptake rates (227 ± 90 mmol C m^−2^ y^−1^ to 520 ± 102 mmol C m^−2^ y^−1^; Table 2) are driven by those due to the changes in DOC control (2.0 ± 36 mmol C m^−2^ y^−1^ to 3.9 ± 35 mmol C m^−2^ y^−1^; Table 2) and in temperature control (225 ± 78 mmol C m^−2^ y^−1^ to 515 ± 92 mmol C m^−2^ y^−1^; Table 2). This is translated into the changes in temperature control driving the changes in DOC uptake rates almost exclusively. Regionally, the changes in DOC uptake rates of free-living bacteria are the largest in the low latitudes (1,013 ± 522 mmol C m^−2^ y^−1^ to 2,242 ± 609 mmol C m^−2^ y^−1^; Table 2) and the lowest in the Southern Ocean (43 ± 25 mmol C m^−2^ y^−1^ to 154 ± 28 mmol C m^−2^ y^−1^; Table 2). The changes in DOC uptake rates are also driven almost exclusively by the changes in temperature control in most regions, similar to the global patterns. By contrast, in the Southern Ocean, the changes in DOC uptake rates (43 ± 25 mmol C m^−2^ y^−1^ to 154 ± 28 mmol C m^−2^ y^−1^; Table 2) are mostly due to the changes in DOC control (34 ± 22 mmol C m^−2^ y^−1^ to 109 ± 22 mmol C m^−2^ y^−1^; Table 2), while the changes in temperature control (7.7 ± 3.6 mmol C m^−2^ y^−1^ to 28 ± 4.7 mmol C m^−2^ y^−1^; Table 2) account for 18–20% of the DOC uptake-rate changes.

**Table 2:** The first-order Taylor decomposition of changes in DOC uptake rates of free-living bacteria under different climate change scenario.

Region	Global ocean	N.H. (>50°N)	N.I. (30–50°N)	L.L. (30°S–30°N)	S.I. (30–50°S)	S.O. (<50°S)
Area in %		11	8	39	14	28
*n*	61,425	6,729	4,814	24,070	8,566	17,246
$\frac{\partial \mu_{\mathrm{DOC}}}{\partial t}$ (i.e., changes in DOC uptake rates, mmol m^−2^ y^−1^)						
SSP1–2.6	227 ± 90	88 ± 16	336 ± 51	1,013 ± 522	276 ± 148	43 ± 25
SSP2–4.5	311 ± 93	103 ± 16	409 ± 52	1,386 ± 548	409 ± 153	77 ± 26
SSP3–7.0	371 ± 97	106 ± 16	430 ± 53	1,648 ± 574	535 ± 159	114 ± 27
SSP5–8.5	520 ± 102	180 ± 17	608 ± 55	2,242 ± 609	705 ± 168	154 ± 28
$\mu_{\max}\frac{\partial }{\partial t}\left(\frac{DOC^{3}}{DOC^{3}+X_{\mathrm{DOC}}^{3}}\right)Q_{10}^{\frac{T-10}{10}}\;$ (i.e., changes in DOC uptake rates driven by DOC changes, mmol m^−2^ y^−1^)						
SSP1–2.6	2.0 ± 36	0.31 ± 12	1.0 ± 23	2.5 ± 75	0.98 ± 76	34 ± 22
SSP2–4.5	2.5 ± 36	0.28 ± 12	1.0 ± 23	3.0 ± 75	1.7 ± 74	58 ± 22
SSP3–7.0	3.0 ± 35	0.27 ± 12	1.1 ± 23	3.3 ± 74	2.5 ± 72	84 ± 23
SSP5–8.5	3.9 ± 35	0.29 ± 11	1.4 ± 23	4.6 ± 74	3.3 ± 70	109 ± 22
$\mu_{\max}\frac{\partial }{\partial t}Q_{10}^{\frac{T-10}{10}}\left(\frac{DOC^{3}}{DOC^{3}+X_{\mathrm{DOC}}^{3}}\right)\;$ (i.e., changes in DOC uptake rates driven by temperature changes, mmol m^−2^ y^−1^)						
SSP1–2.6	225 ± 78	88 ± 10	335 ± 44	1,010 ± 513	275 ± 122	7.7 ± 3.6
SSP2–4.5	308 ± 82	103 ± 10	407 ± 45	1,382 ± 538	407 ± 129	14 ± 3.8
SSP3–7.0	368 ± 86	106 ± 11	428 ± 46	1,643 ± 564	532 ± 136	20 ± 4.2
SSP5–8.5	515 ± 92	179 ± 12	606 ± 47	2,235 ± 600	701 ± 147	28 ± 4.7

Errors as standard errors.

## 4. Discussion

### 4.1. Model skill assessment

We first discuss the credibility of the future bacterial projections using the skill scores against the available observations at different temporal and spatial coverages, despite the challenges associated with the general sparsity of the bacterial measurement data. The model shows better skill for capturing the yearly biomass averages compared to the monthly biomass averages. The interannual variability is not captured everywhere, which however should be less of concern given that it is bacterial biomass and production averaged over the first and last 23 years for each SSP period that serve as the basis for our analysis. The major challenge in the analysis of the observational data is to estimate bacterial carbon biomass and production from abundance and cell-specific thymidine incorporation rates using the appropriate carbon conversion factors. No universally-accepted carbon conversion factors exist for converting cell count to bacterial carbon biomass and radioisotope incorporation to bacterial production, and most microbial ecologists arbitrarily choose these conversion factors based on literatures rather than *via* experimental determination. For instance, bacterial carbon biomass estimation by MAREDAT global database adopts a single carbon conversion factor of 9.1 fg C cell^−1^ (Buitenhuis et al., 2012), which is slightly lower than the value known to be more suitable for open oceans (10 fg C cell^−1^) and therefore used for BATS and HOT data sets in our analysis (Ducklow and Carlson, 1992; Caron et al., 1995). Moreover, the carbon content of bacteria varies under varying growth conditions (Ducklow, 2000), thereby seasonally and geographically. The carbon content of bacteria in open oceans also varies considerably, ranging from 5.5 to 23.5 fg C cell^−1^ (Buitenhuis et al., 2012). All these together contribute to relatively poor model biomass skill at global scale compared to regional scale.

The model-data discrepancy for bacterial production can be explained in a similar manner. It has been demonstrated that the thymidine conversion factor varies much more greatly than the conversion factors for other types of radioisotope incorporation rates to bacterial production (e.g., ^3^H-leucine incorporation; Ducklow, 2000), showing 4–10 fold differences between the minimum and maximum values even at the same sampling location. For instance, the thymidine conversion factor ranges from production of 0.5 × 10^18^ cells to 4.9 × 10^18^ cells per mol of ^3^H-thymidine incorporation in Delaware Bay and from production of 0.8 × 10^18^ cells to 3.4 × 10^18^ cells per mol of ^3^H-thydimine incorporation in the subarctic Pacific (Kirchman, 1992). The fact that our thymidine conversion factor lies within these reported variations is probably a main driver of the model overestimation of bacterial production, as shown by model overestimation of bacterial production by 3.6 times (Supplementary Table 2). Using the above minimum and maximum thymidine conversion factors, the model overestimates bacterial production by 0.6 times and 5.9 times, respectively. However, a recent study on phospholipid turnover rates suggests that the ^3^H-thydimine incorporation method underestimates bacterial growth rates by ~3.8 times at BATS (Popendorf et al., 2020) because actively growing bacteria in the Sargasso Sea do not take up as much exogeneous thymidine as phosphate (Longnecker et al., 2010). If this is the case, the BP data from BATS are also underestimated and in fact similar to the simulated bacterial production rates, adding confidence to our model skill.

The model overestimation of bacterial production is associated with high values of the simulated BGE (i.e., ~0.44; Table 1) compared to those from laboratory and field studies. del Giorgio and Cole (2000) demonstrate that the mean BGE values tend to increase from open to estuarine waters (i.e., 0.15 ± 0.12 from 62 measurements in open oceans, 0.27 ± 0.18 from 123 measurements in coastal oceans, 0.37 ± 0.15 from 54 measurements in estuaries). Other BGE estimates include 0.24 ± 0.10 in the Arctic Kara Sea (Meon and Amon, 2004) and 0.24 ± 0.13 in the Antarctic Ross Sea (Carlson et al., 1999). Similar BGE values have been reported in lower-latitude systems (del Giorgio and Cole, 2000). However, studies with higher BGE values (e.g., ~0.5) than our BGE value are not unprecedented (Ducklow and Carlson, 1992; Jahnke and Craven, 1995; del Giorgio et al., 1997; del Giorgio and Cole, 2000). One may also argue that it is unrealistic to have consistent BGE values across different regions and climate scenarios, but a compilation of studies suggests that there is no clear evidence that BGE varies significantly with temperature or systematically among different regions such as the North Atlantic, the Equatorial and Subarctic Pacific, the Western Arctic, and Ross Sea (del Giorgio and Cole, 1998; López-Urrutia and Morán, 2007; Vázquez-Domínguez et al., 2007; Alonso-Sáez et al., 2008; Kirchman et al., 2009).

### 4.2. Global trends

At least three factors can drive the projected changes in bacterial biomass: the changes in (a) physical mixing and circulation that can lead to lateral or vertical transport of bacteria; (b) the net growth (i.e., bacterial production); and (c) the loss of biomass through grazing and mortality. We cannot quantitatively differentiate the given biomass changes into the contribution by each of these three components because the loss terms were not saved during CMIP6 experiments. We hypothesize that the loss of bacterial biomass due to physical transport may not increase significantly, given that CMCC-ESM2 simulates an increase in stratification over the next century (Lovato et al., 2022) similar to most CMIP6 models (Kwiatkowski et al., 2020). This then makes the competition between the net growth and loss of biomass by grazing and mortality a major driver of the biomass changes. In turn, temperature and organic matter concentrations regulate the loss of biomass (Eqs. 9 and 10) and net growth (Eqs. (11) and (12)) through organic matter uptake (see “Discussion 4.4. Underlying mechanisms for details).

One pattern in common between the global and regional composites is that except for the Southern Ocean, warming drives the net biomass declines despite increasing bacterial production. This suggests that the warming-induced increases in the loss terms (i.e., grazing, mortality) exceeds those in the source term (i.e., bacterial production), or that BGE is decreasing because of warming. While the effects of climate change on the loss terms are poorly characterized at present, several studies have suggested that warming alters the abundance, trophic activities, and grazing rates of microzooplankton (Rose et al., 2009; Chen et al., 2012; Caron and Hutchins, 2013). As shown above, BGE is consistent across different regions and SSPs. This final BGE “equilibrium” achieved in the model simulation is mainly dependent on the value of the activity respiration fraction (i.e., 0.5, Lovato et al., 2022) that represents the main carbon loss, while the rest of the variation is attributed to the changes in other rate terms dependent on the same temperature parameterization for bacteria. Given that the dissolved fraction of the total organic carbon pool determines the amount of free-living biomass (Eq. 3), it is the magnitude of changes in each organic carbon pool as well as total bacterial biomass that drives the percentage declines in free-living and particle-attached biomasses in response to warming. Globally, warming results in large declines in POC concentrations (5–10%), while DOC concentrations increase by similar percentages (4–8%), resulting in lower biomass declines for free-living cells compared to particle-attached cells (Table 1). These patterns are consistent with other studies that the cumulative effects of the climate change-driven alterations in ocean physics and biogeochemistry may induce the net reductions in POC flux out of the surface ocean (Bopp et al., 2001, 2013; Boyd, 2015; Laufkötter et al., 2016) and in the downward flux of DOC (Lønborg et al., 2020), while global annual mean NPP increases slightly under climate change for this model (Lovato et al., 2022). In addition, rising temperature and nutrient limitation of autotrophic growth have been shown to increase the partitioning from POC to DOC pools *via* an increase in the percentage of DOC (Myklestad and Haug, 1972; Biddanda and Benner, 1997; Engel et al., 2002, 2011; Wohlers et al., 2009; Kim et al., 2011; Vernet et al., 2017), supporting our results for the DOC increases and POC decreases and, in turn, the free-living and particle-attached biomass changes.

### 4.3. Regional trends and variability

The classic latitudinal breakdowns used for the regional composites reveal significant spatial variability in bacterial dynamics, suggesting strong coupling between bacteria and other ecological variables (e.g., NPP) because of coherent physical and environmental forcings. This suggests that the model organic carbon dynamics reproduce the observed, contemporaneous trophic relationship between bacteria and phytoplankton mediated *via* phytoplankton-derived labile DOC (Lancelot, 1979; Norrman et al., 1995), though phytoplankton are not the only organism that produce labile DOC.

Our results demonstrate that the most distinct responses arise from the Southern Ocean in which warming induces total biomass increases, in contrast to other regions that experience the net total biomass declines. This is largely driven by the two patterns unique to the Southern Ocean: (a) the particle-attached biomass declines are not as large and (b) it is the only region where the free-living biomass increases. The fact that particle-attached cells are simulated to be a major fraction of the bulk bacterial community in the Southern Ocean also contributes to the opposite biomass trends. Studies have demonstrated that particle-attached bacteria are mainly influenced by particle quality and availability (Yung et al., 2016) and do not respond as sensitively to temperature as free-living bacteria (Bachmann et al., 2018) because detrital particles that bacteria are attached to act as a buffer and diminish their exposure to surrounding water temperature (Lyons et al., 2010; Yung et al., 2016). Particle-attached bacteria are also thought to be copiotrophic as they are more adaptable to variable substrate concentrations and capable of rapid growth under nutrient-rich conditions compared to free-living bacteria (Lauro et al., 2009). All these factors would justify for the cold Southern Ocean waters to hold a higher particle-attached portion, although the model simulates such patterns for different reasons. In the Southern Ocean, the model simulates higher POC but lower DOC than other regions (Table 1), which then together increases the partitioning of total bacterial biomass into more particle-attached biomass and less free-living biomass to begin with (Eqs. 3 and 4). Under climate change, the increases in both organic matter pools lead to the increases in free-living biomass and only slight decreases in particle-attached biomass. Because of the larger increases in DOC than POC, the fraction of POC decreases (Eq. 4) and thus results in the decreases in particle-attached biomass. The Southern Ocean is also distinct that bacterial carbon demand, respiration, and production increase to a larger extent under warming. This suggests the greater impacts of the warming-induced increases in bacterial metabolism in the Southern Ocean, otherwise significantly suppressed than other regions (Table 1). These patterns are consistent with the increases in bacterial stocks and rates in response to future warming along the West Antarctic Peninsula simulated by data-assimilative models (Kim et al., 2021, 2022).

By contrast, the bacterial responses to warming in the northern high latitudes contradict almost every pattern emerging from the Southern Ocean, despite both being high-latitude, polar oceans. The largest warming-driven declines in bacterial biomass are simulated for the northern high latitudes exceeding their respective global average declines, as well as resulting in these regions no longer exhibiting the largest biomass under warming. A decadal study in the temperate (~15.8°C) North Atlantic has shown that ocean warming drives a slight reduction in total bacterial biomass and a large shift to small, slowly growing low nucleic acid bacterial cells at least in spring seasons (Morán et al., 2015). However, we do not have data to compare the model results with because year-round studies are lacking in these regions. The large, simulated biomass declines are in part because of the bacterial production declines in 2076–2099 compared to 1990–2013 for higher SSPs unique to the northern high latitudes, while the rest is attributed to the increased biomass loss through grazing and mortality under warming. The bacterial production declines are, in turn, due to the changes in DOC uptake rates and free-living biomass (Eq. (11)), the effects of which cannot be assessed due to the large residual from the first-order Taylor decomposition (not shown). Compared to the Southern Ocean, DOC concentrations are much higher and POC concentrations are much lower in the northern high latitudes (Table 1). Warming further amplifies such differences *via* large POC decreases but only slight DOC increases, resulting in stronger partitioning to free-living bacterial biomass (Eqs. 3 and 4). Such differences in the warming-mediated changes of organic substrates partly explain why the northern high latitudes show particularly large declines in particle-attached biomass.

Distinct to the low latitudes is that regardless of warming, bacterial rates are high but stocks are low relative to other regions. Observations along the north–south Atlantic transects have shown that compared to higher latitudes, large areas in the low-latitude oceanic gyres are characterized by high bacterial metabolism *via* the elevated ratios of the bacterial carbon demand to NPP and the ratio of bacterial production to NPP (Hoppe et al., 2002). These patterns are partly due to low NPP in oligotrophic low-latitude waters, but bacterial metabolic rates also tend to be higher in the warm subtropical Atlantic (Alonso-Sáez et al., 2007). Nevertheless, warming in the low-latitude regions probably has greater or as great impacts on grazing and mortality (Morán et al., 2017) as on bacterial production, resulting in rather low average biomass compared to other regions (Table 1). It has been suggested that the effect of ocean warming on microbial stocks tends to be greater at middle to high latitudes compared to the tropics (Ward, 2015), so bacteria may not be an exception to this (Morán et al., 2017). Morán et al. (2017) has demonstrated that 1°C warming causes the reduction in bacterial biomass in warm waters (>26°C), the range of temperature equivalent to the low latitudes in our study.

### 4.4. Underlying mechanisms of changes in DOC uptake rates of free-living bacteria

It should be noted that substrate uptake rates are a function of temperature as well as biomass for particle-attached bacteria that is, in turn, affected by the competition between bottom-up and top-down controls. Top-down control changes with temperature (e.g., grazing and mortality rates). Thus, bottom-up and top-down controls are inherently coupled to each other, and most studies aiming to determine a dominant control on bacteria in fact fail to truly separate the effects of these different factors from one another. The similar argument holds for mechanisms controlling bacterial production. Conventionally, temperature control on bacterial production is assessed using an Arrhenius-type relationship (Westrich and Berner, 1988; Lønborg et al., 2016) given by: $BP=BP_{0}\times e^{–E_{a}/R\times T}$ , where *E_a_* is an apparent activation energy (J mol^−1^), *BP* is bacterial production, *BP*_0_ is the theoretical bacterial production in the absence of *E_a_*, *R* is the universal gas constant (*R* = 8.314 J mol^−1^ K^−1^), and *T* is the absolute temperature (K). *E_a_* (kJ mol^−1^) is estimated as the absolute value of the regression slope in an Arrhenius plot (i.e., *y*-variable as a natural logarithm of bacterial production onto *x*-variable as 1/*T*) multiplied by the universal gas constant. However, this approach does not separate the direct impact of temperature change from the impacts of other temperature-mediated changes including biomass and organic carbon concentrations that bacterial production covaries with (Kirchman et al., 2009). As an alternative, we utilize the Taylor decomposition to separate the impacts of the different controlling mechanisms and how each of these shapes the bacterial responses to ocean warming.

The results from the Taylor decomposition (Eq. (13)) suggest at least two apparent regional patterns for the free-living bacterial responses to warming: (a) in the northern high and low latitudes, the increases in temperature control are a main driver of the increases in DOC uptake rates; and (b) in the Southern Ocean, the increases in DOC control are a main driver of the increases in DOC uptake rates of free-living bacteria. DOC in surface waters of the Southern Ocean waters is known to be dominated by refractory material upwelled from the deep ocean and also the lowest of the world oceans (~50 μM, Hansell, 2013) because of low NPP. The model also simulates the lowest DOC values in the Southern Ocean (Table 1). As a result, assessing substrates is a major constraint for bacterial growth (Church et al., 2000), and the supply of labile DOC by phytoplankton usually enhances bacterial metabolism in the Southern Ocean (Christaki et al., 2014). A switch in the modes of control has been proposed by Morán et al. (2017) that temperature does not exert a significant control on bacterial production when bacteria are severely limited by DOC and that temperature can only take over control upon an alleviation of DOC limitation. This observation is traced back to Pomeroy Hypothesis where temperature limitation of bacteria is relieved by increased organic matter availability (Pomeroy and Deibel, 1986; Pomeroy et al., 1990, 1991; Wiebe et al., 1992). These studies support our findings for the Southern Ocean such that under warming, increasing DOC poses a greater impact on increasing DOC uptake rates than increasing temperature itself because bacteria still might be under DOC stress even after warming.

Then what makes the effect of temperature control greater than that of DOC control in the northern high and low latitudes? Bacteria may not be under as strong DOC limitation as in the Southern Ocean, given much higher DOC values in these regions (Table 1). Unlike the Southern Ocean, significant fluvial and terrigenous sources provide allochthonous carbon material and increase total DOC in the Arctic Ocean, as simulated in the model using the climatological riverine discharges of organic carbon substrates (Mayorga et al., 2010). Most of fluvial DOC might not be as degradable by bacteria as labile DOC (Hansell et al., 2004; Engel et al., 2019), but there is also evidence of the rapid removal of old, fluvial DOC upon its release to the Arctic shelves (Hansell et al., 2004; Letscher et al., 2011). In the tropics, DOC concentrations are higher than other regions because strong stratification of the upper water column drives the accumulation of DOC that is resistant to bacterial degradation (Hansell et al., 2009). Thus, the switch behavior in the modes of control from DOC to temperature (Morán et al., 2017) could be applied in the northern high and low-latitude regions. In these regions, bacteria are not limited by DOC, so temperature can take over control on bacterial metabolism. Other studies also have shown that the Arctic warming leads to increased bacterial activities (Middelboe and Lundsgaard, 2003; Kirchman et al., 2005), presumably through the increased organic carbon bioavailability from warming that modulates and amplifies the effect of elevated temperature (Piontek et al., 2015).

## 5. Conclusion

This study analyzes the future responses of marine heterotrophic bacteria to century-scale future climate change, explores the underlying drivers of the simulated responses, and raises issues about the different climate-driven dynamics of free-living versus particle-attached bacteria and the interplay of organic substrate and temperature limitation of bacterial growth. A large challenge arises for bacterial modeling studies because the measurement data on bacterial stocks and rates are scarce in the ocean compared to those for other biogeochemical variables (e.g., NPP, nutrients, chlorophyll, DOC, and POC), leading to rather limited model validation efforts. The effect of the uncertainties in bacterial carbon conversion factors is also not trivial. Other challenges concern the treatment of bacteria in mechanistic models, in particular choices on the level of detail for microscopic and molecular processes. Despite serving as one of the very few bacteria-resolving global models, our model does not simulate important processes, such as bacterial evolution and adaptation to climate change, particle size or characteristics that adjust the behavior of particle-attached bacteria, and the relationship between particle-attached densities and the efficiency of signaling and quorum sensing (Gram et al., 2002). The scope of our study is also limited to upper 100 m layer, whereas most attenuation of sinking organic matter by bacteria occurs in the mesopelagic zone. Further studies should focus on deep-sea bacterial dynamics using the model results below 100 m and the data sets on deep-ocean prokaryote abundance and metabolic rates (e.g., Herndl et al., 2022).

Keeping such limitations in mind, this study serves as an early effort to systematically quantify the climate-driven changes in bacterial dynamics at global scales. Compared to numerous previous studies, our study also emphasizes the need for teasing out the effects of temperature and DOC control from each other, challenging the conventional views in microbial oceanography. Bacteria unarguably play an important role in the ocean carbon cycle by decreasing the amount of organic carbon exported to the deep ocean, both through free-living and particle-attached cells, but more directly so by particle-attached cells that respire more than half of the sinking organic carbon to CO_2_ (i.e., BGE = 44%, Table 1). Our study suggests that under the most severe warming scenario, bacteria may respire up to 15% more globally and 21% more in the Southern Ocean (Table 1, Figures 2, 4), consistent with the initial hypothesis for global warming impacts on bacteria (Pomeroy et al., 1991). Provided that bacteria account for most heterotrophic community respiration in open oceans (Williams, 1981; Rivkin and Legendre, 2001), our study proposes a potentially important bacterial feedback to the functioning of the biological carbon pump and partitioning of organic carbon pools between surface and deep layers.

## Data availability statement

The data sets (2021) used for this study can be found under Cmip6 Data Search tool in the ESGF website.

## Author contributions

HK analyzed the model results and wrote the manuscript. CL analyzed and interpreted the model results. TL performed the simulations and provided the model results. SD and HD interpreted the model results and contributed to writing the discussion part of the manuscript. All authors contributed to the article and approved the submitted version.

## Funding

This is the NSF Center for Chemical Currencies of a Microbial Planet (C-CoMP) publication #019. HK was supported by C-CoMP (NSF Award 2019589) and the Independent Research & Development Award Program at Woods Hole Oceanographic Institution. CL acknowledges support from the Swiss National Science Foundation under grant 174124. TL was supported by CRESCENDO EU project that funded model development for MIP6. SD was supported by C-CoMP.

## Conflict of interest

The authors declare that the research was conducted in the absence of any commercial or financial relationships that could be construed as a potential conflict of interest.

## Publisher’s note

All claims expressed in this article are solely those of the authors and do not necessarily represent those of their affiliated organizations, or those of the publisher, the editors and the reviewers. Any product that may be evaluated in this article, or claim that may be made by its manufacturer, is not guaranteed or endorsed by the publisher.

## Supplementary material

The Supplementary material for this article can be found online at: https://www.frontiersin.org/articles/10.3389/fmicb.2023.1049579/full#supplementary-material. The data sets (2021) for this study can be found in the ESGF (https://esgf-654%2node.llnl.gov/projects/esgf-llnl/).
