# Supplementary material for: Projected 21st-century changes in marine heterotrophic bacteria under climate change
Source: Front Microbiol. 2023 Feb 16;14:1049579. doi: 10.3389/fmicb.2023.1049579 (PMC9978487; doi:10.3389/fmicb.2023.1049579)
Supplement: Supplementary file 1 [file Data_Sheet_1.docx]

Supplementary Material

**Supplementary Table 1. Institution, source, experiment, and variant IDs, integration time, and variable names analyzed in the present study.** The files containing the variables, baccupt and baccres, are provided by CMCC ESM team, while those for the remaining variables are available publicly on ESGF servers under the CMIP6 project. bacc: bacterial carbon biomass, baccupt: bacterial organic carbon uptake, baccres: bacterial respiration, dissoc: DOC, detoc: POC, and thetao: water temperature.

| **Institution ID** | **Source ID** | **Experiment ID** | **Variant ID** | **Integration time** | **Variables** |
| --- | --- | --- | --- | --- | --- |
| CMCC | CMCC-ESM2 | historical | r1i1p1f1 | 1850-2014 | bacc baccupt  baccres  dissoc  detoc  thetao |
| CMCC | CMCC-ESM2 | ssp126 | r1i1p1f1 | 2015-2100 |  |
| CMCC | CMCC-ESM2 | ssp245 | r1i1p1f1 | 2015-2100 |  |
| CMCC | CMCC-ESM2 | ssp370 | r1i1p1f1 | 2015-2100 |  |
| CMCC | CMCC-ESM2 | ssp585 | r1i1p1f1 | 2015-2100 |  |

**Supplementary Table 2. Skill assessment for bacterial carbon biomass and production (1988-2011, upper 100 m).** MAREDAT: MARine Ecosystem biomass DATa (compiled from a total of 9,284 data points over 1° × 1° × 33 vertical layers × 12 months; Buitenhuis et al., 2012). BATS: the Bermuda Atlantic Time-series Study. HOT: the Hawaii Ocean Time-series (HOT) at Station ALOHA. RMSD: total root-mean-square distance. *n*: sample size. n/s for not a significant correlation (*p* > 0.05).

| **Model skill scores** | **Bacterial biomass (mmol m^-2^)** | | | | | **Bacterial production (mmol m^-2^ d^-1^)** |
| --- | --- | --- | --- | --- | --- | --- |
|  | **MAREDAT** | **BATS** | | **HOT** | | **BATS** |
|  | **Monthly** | **Monthly** | **Yearly** | **Monthly** | **Yearly** | **Yearly** |
| Model mean | 31.8 | 27.6 | 32.7 | 23.1 | 25.0 | 12.8 |
| Observed mean | 49.2 | 32.5 | 32.9 | 24.9 | 24.4 | 3.6 |
| Model standard deviation | 11.5 | 10.0 | 0.63 | 5.7 | 2.4 | 0.43 |
| Observed standard deviation | 16.4 | 8.9 | 5.0 | 2.6 | 3.9 | 1.8 |
| Correlation coefficient | 0.24 | 0.38 | n/s | n/s | n/s | n/s |
| RMSD | 24.7 | 11.6 | 4.9 | 6.4 | 5.0 | 9.4 |
| Average bias | -17.4 | -4.9 | -0.24 | -1.8 | 0.63 | 9.2 |
| Average absolute error | 20.5 | 9.5 | 3.5 | 5.8 | 4.1 | 9.2 |
| Centered RMSD | 17.5 | 10.5 | 4.9 | 6.1 | 5.0 | 1.8 |
| Reliability index | 1.8 | 1.5 | 1.2 | 1.3 | 1.2 | 4.5 |
| *n* | 98 | 54 | 23 | 8 | 21 | 22 |

**Supplementary Table 3. Parameter choice, values, unit, and references for BFM bacterial formulations.** Details on the parameter values used in Eqs. 1-10 of the main text. References cited: Vichi, M., Masina, S., & Navarra, A. (2007a). A generalized model of pelagic biogeochemistry for the global ocean ecosystem. Part II: Numerical simulations. Journal of Marine Systems, 64(1), 110-134; Zimmerman, A. E., Allison, S. D., & Martiny, A. C. (2014). Phylogenetic constraints on elemental stoichiometry and resource allocation in heterotrophic marine bacteria. Environmental microbiology, 16(5), 1398-1410; Lovato, T., Peano, D., Butenschön, M., Materia, S., Iovino, D., Scoccimarro, E., et al. (2022). CMIP6 simulations with the CMCC Earth System Model (CMCC-ESM2). Journal of Advances in Modeling Earth Systems, 14, e2021MS002814.

| **Parameters** | **Definition** | **Unit** | **Values** | **References** |
| --- | --- | --- | --- | --- |
| $\text{Q}_{\text{10}}^{\text{B}}$ | Temperature limitation factor for bacteria | Unitless | 2.95 | Vichi et al. (2007a) |
| $\text{μ}_{\text{max}}$ | Maximum specific uptake rate of organic carbon | d^-1^ | 3.0 | Zimmerman (2013) |
| $\text{X}_{\text{DOC}}$ | Half-saturation coefficient of DOC | mmol C m^-3^ | 83.3 | Lovato et al. (2022) |
| $\text{X}_{\text{POC}}$ | Half-saturation coefficient of POC | mmol C m^-3^ | 4.17 | Lovato et al. (2022) |
| $\text{z}_{\text{0}}$ | Reference depth for depth limitation factor of bacteria | m | 400 | Lovato et al. (2022) |
| $\text{b}$ | Exponent of depth limitation factor of bacteria | Unitless | -1.25 | Lovato et al. (2022) |
| $\text{b}_{\text{B}}$ | Respiration rate of bacteria | d^-1^ | 0.01 | Vichi et al. (2007a) |
| $\text{γ}_{\text{B}}^{\text{a}}$ | Fraction of active respiration of bacteria | Unitless | 0.5 | Vichi et al. (2007a) |
| $\text{γ}_{\text{B}}^{\text{o}}$ | Fraction of additional respiration of bacteria under low oxygen conditions | Unitless | 0.1 | Vichi et al. (2007a) |
| $\text{h}_{\text{B}}^{\text{o}}$ | Half-saturation coefficient for oxygen regulating factor of bacteria | mmol O m^-3^ | 15 | Vichi et al. (2007a) |
| $r_{Z}^{0}$ | Potential specific growth rate of microzooplankton | d^-1^ | 3.0 | Lovato et al. (2022) |
| $\text{Q}_{\text{10}}^{\text{Z}}$ | Temperature limitation factor for microzooplankton | Unitless | 2.0 | Vichi et al. (2007a) |
| $\text{δ}_{\text{Z,B}}$ | Feeding affinity of microzooplankton on bacteria | Unitless | 0.2 | Lovato et al. (2022) |
| $\text{h}_{\text{Z}}^{\text{F}}$ | Total food ingestion potential of microzooplankton | mmol C m^-3^ | 0.02 | Vichi et al. (2007a) |
| $\text{μ}_{\text{Z}}$ | Feeding threshold of microzooplankton | mmol C m^-3^ | 1.67 | Vichi et al. (2007a) |
| $\text{d}_{\text{B}}$ | Linear mortality rate | d^-1^ | 0.05 | Lovato et al. (2022) |

**Supplementary Figure 1. Skill assessment for bacterial carbon biomass (mmol C m^-2^) integrated in the upper 100 m for MAREDAT: a) data and model time-series, b) model-observation misfits over time, c) data and model scatter plots with the 1:1 line, d) model-observation misfits (bias) versus model.** Blue dots: model values and black dots: observations. BB: bacterial carbon biomass.

**Supplementary Figure 2. Skill assessment for bacterial carbon biomass (mmol m^-2^) integrated in the upper 100 m for HOT: a) data and model time-series, b) model-observation misfits over time, c) data and model scatter plots with the 1:1 line, d) model-observation misfits (bias) versus model.** Blue dots: model values and black dots: observations. BB: bacterial carbon biomass.


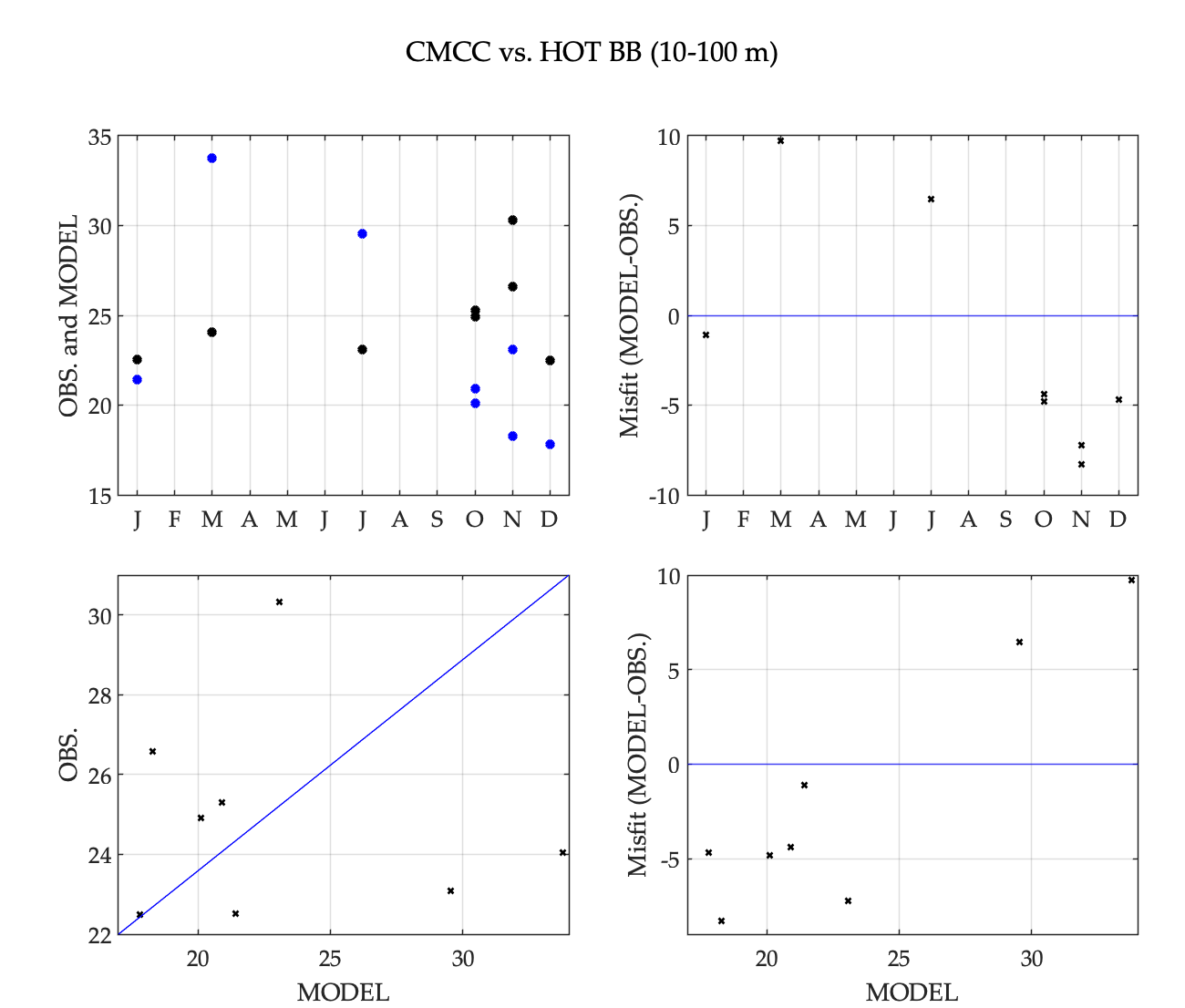

**Supplementary Figure 3. Skill assessment for bacterial carbon biomass (mmol m^-2^) integrated in the upper 100 m for BATS: a) data and model time-series, b) model-observation misfits over time, c) data and model scatter plots with the 1:1 line, d) model-observation misfits (bias) versus model.** Blue dots: model values and black dots: observations. BB: bacterial carbon biomass.


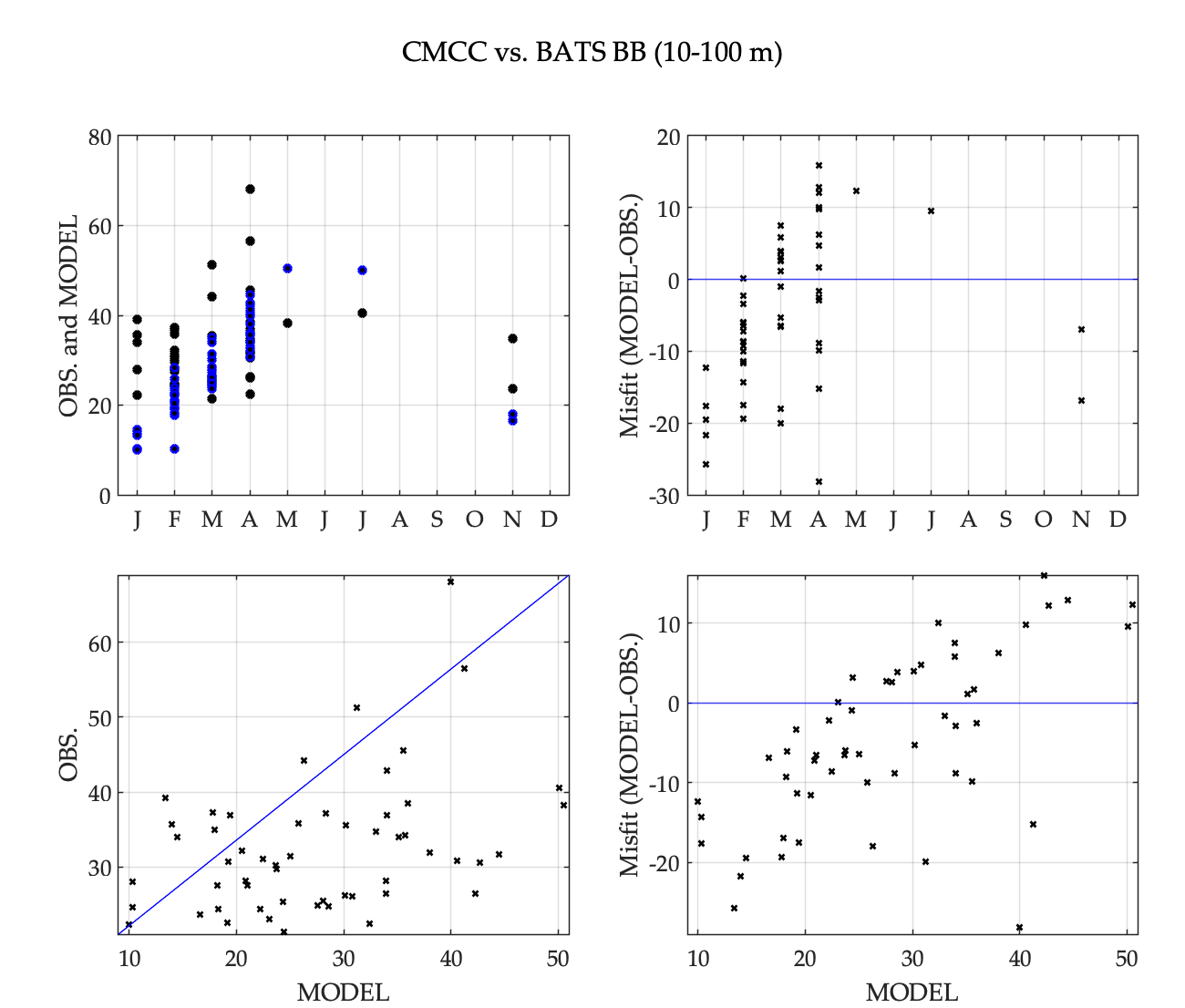

**Supplementary Figure 4. Skill assessment for bacterial production (mmol C m^-2^ d^-1^) integrated in the upper 100 m for BATS: a) data and model time-series, b) model-observation misfits over time, c) data and model scatter plots with the 1:1 line, d) model-observation misfits versus model.** Blue dots: model values and black dots: observations. BP: bacterial production.

**Supplementary Figure 5. Global projections of bacterial carbon biomass under different climate change scenarios (2076-2099).** BB: bacterial carbon biomass, BB_DOC_: biomass of free-living bacteria, BB_POC_: biomass of particle-attached bacteria. All variables are depth-integrated in the upper 100 m. Solid-line contours as standard deviation from averaging over 2076-2099. Baseline period (a-c), SSP1-2.6 (d-f), SSP2-4.5 (g-i), SSP3-7.0 (j-l), and SSP5-8.5 (m-o).


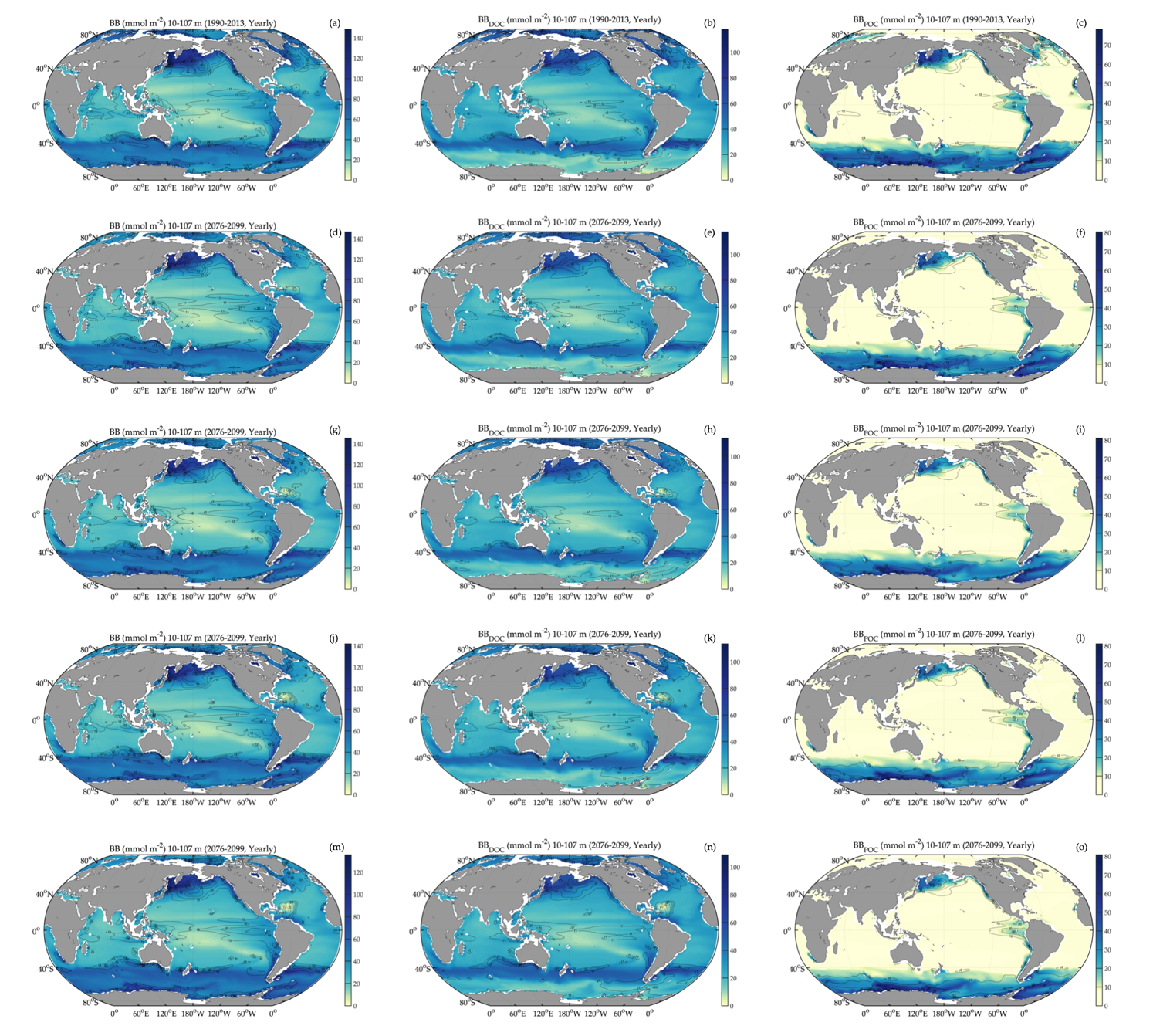


**Supplementary Figure 6. Global projections of bacterial carbon rates under different climate change scenarios (2076-2099).** BCD: bacterial carbon demand, BR: bacterial respiration, and BP: bacterial production. All variables are depth-integrated in the upper 100 m. Solid-line contours as standard deviation from averaging over 2076-2099. Baseline period (a-c), SSP1-2.6 (d-f), SSP2-4.5 (g-i), SSP3-7.0 (j-l), and SSP5-8.5 (m-o).

**
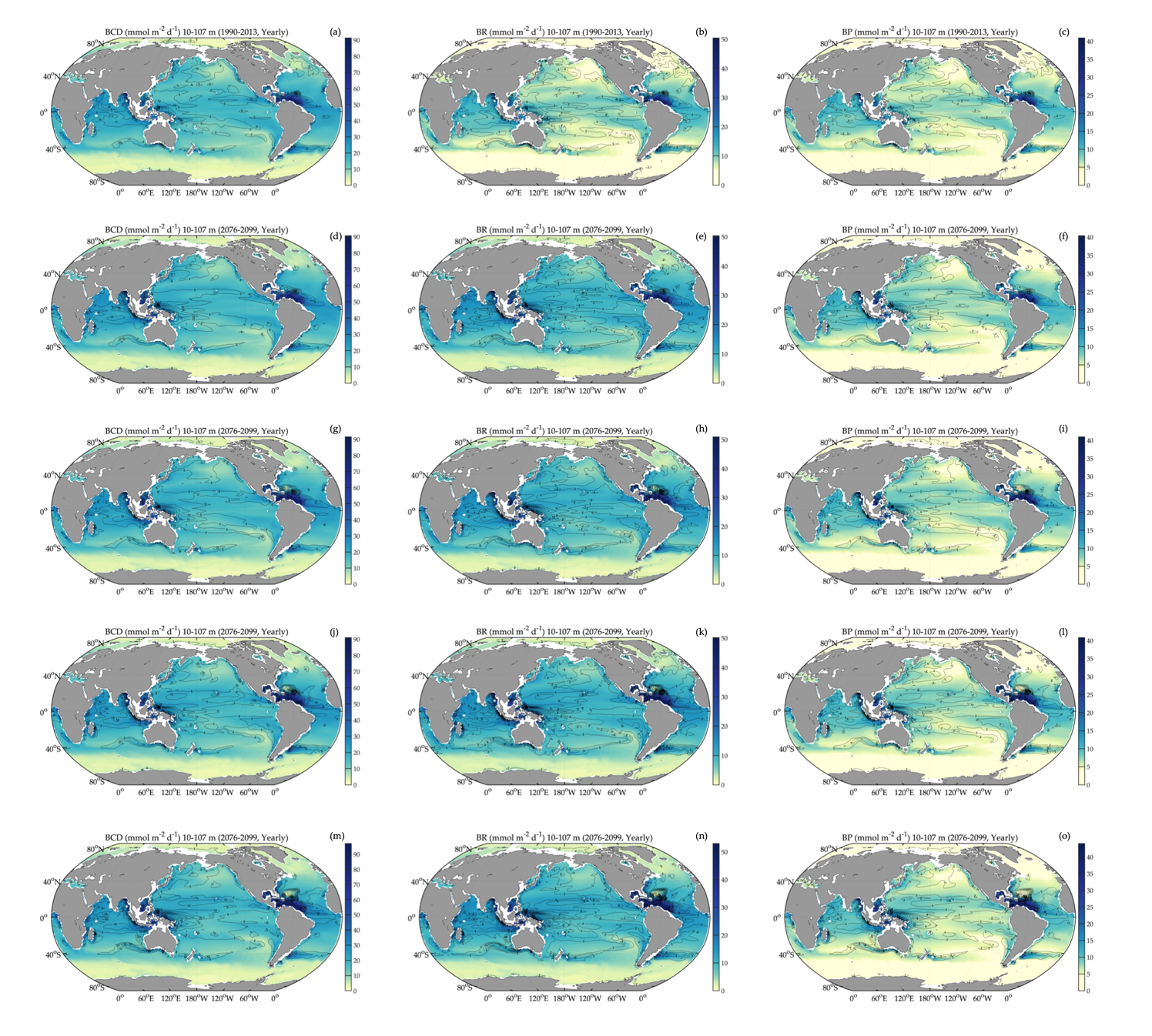
**

**Supplementary Figure 7. Global projections of particulate (POC) and dissolved organic carbon (DOC) and potential water temperature under different climate change scenarios (2076-2099).** POC and DOC are depth-integrated in the upper 100 m, while water temperature is averaged in the upper 100 m. Solid-line contours as standard deviation from averaging over 2076-2099. Baseline period (a-c), SSP1-2.6 (d-f), SSP2-4.5 (g-i), SSP3-7.0 (j-l), and SSP5-8.5 (m-o).


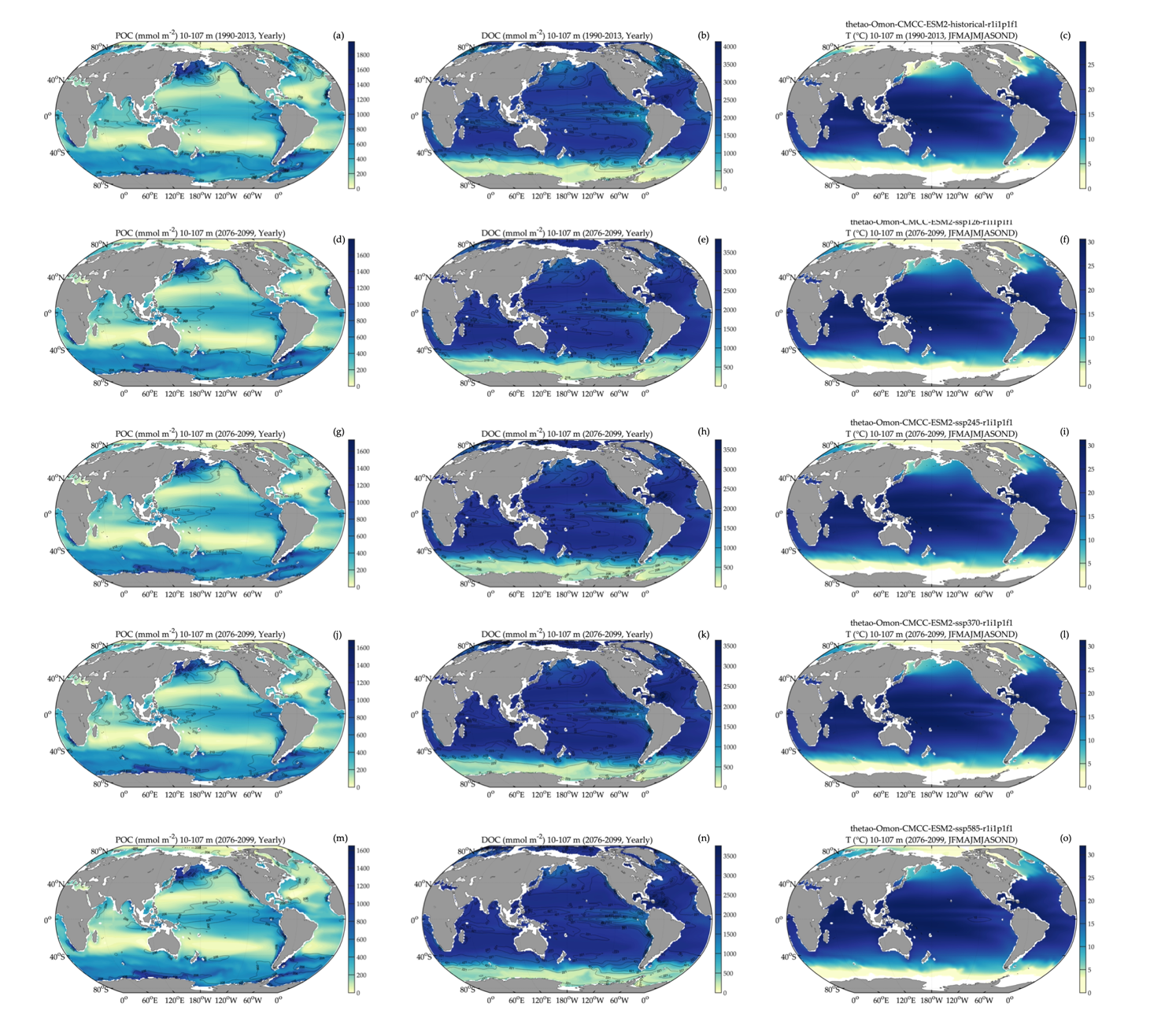


**Supplementary Figure 8. Global projections of POC, DOC, and potential water temperature and their anomalies under different climate change scenarios (2076-2099).** Anomalies are 2076-2099 average values relative to 1990-2013 average values.


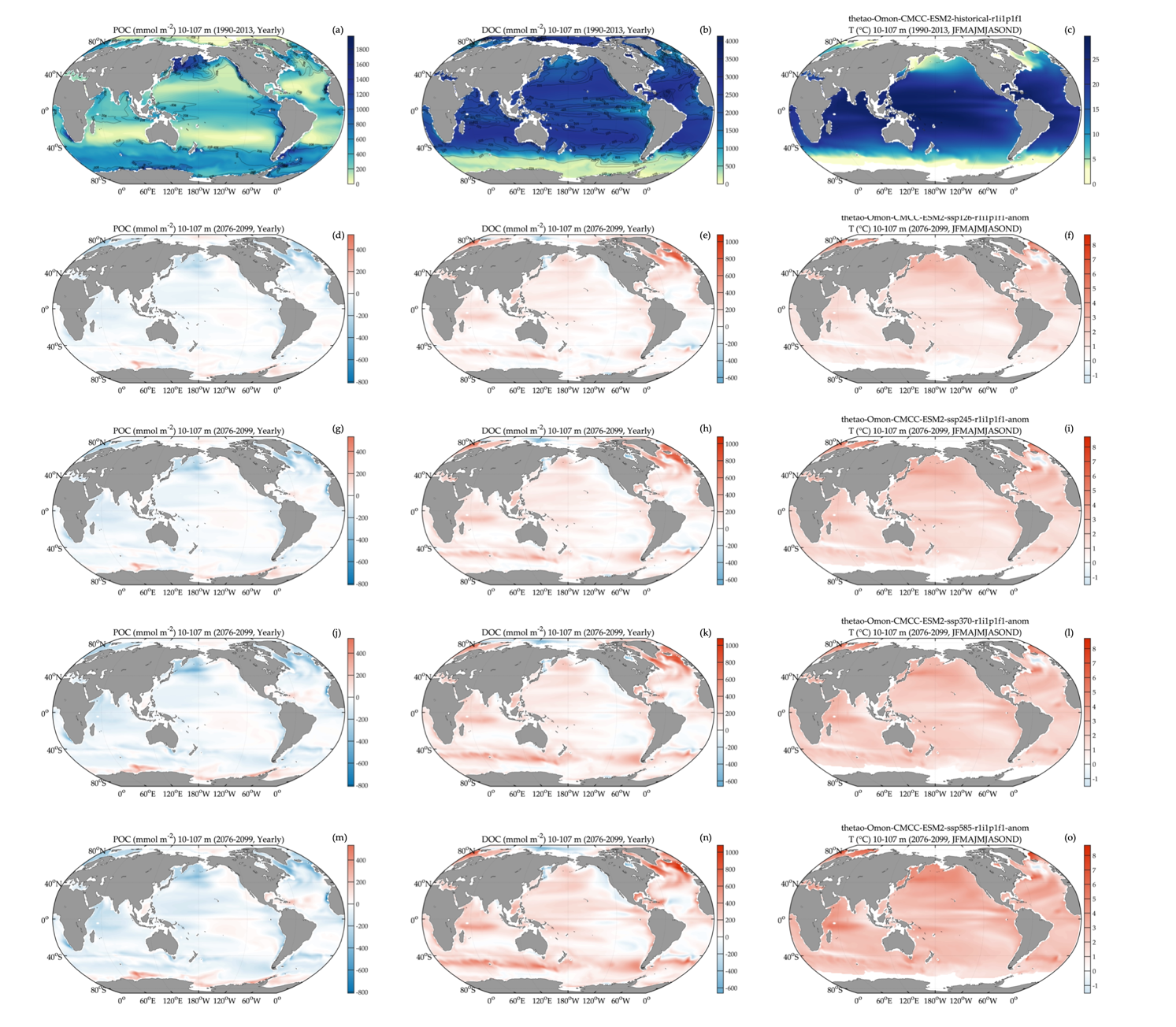


**Supplementary Figure 9. Trends in POC, DOC, and potential water temperature under different climate change scenarios.** N.H.: northern high latitudes, N.I.: northern intermediates, L.L.: low latitudes, S.I.: southern intermediates, and S.O.: Southern Ocean.

**
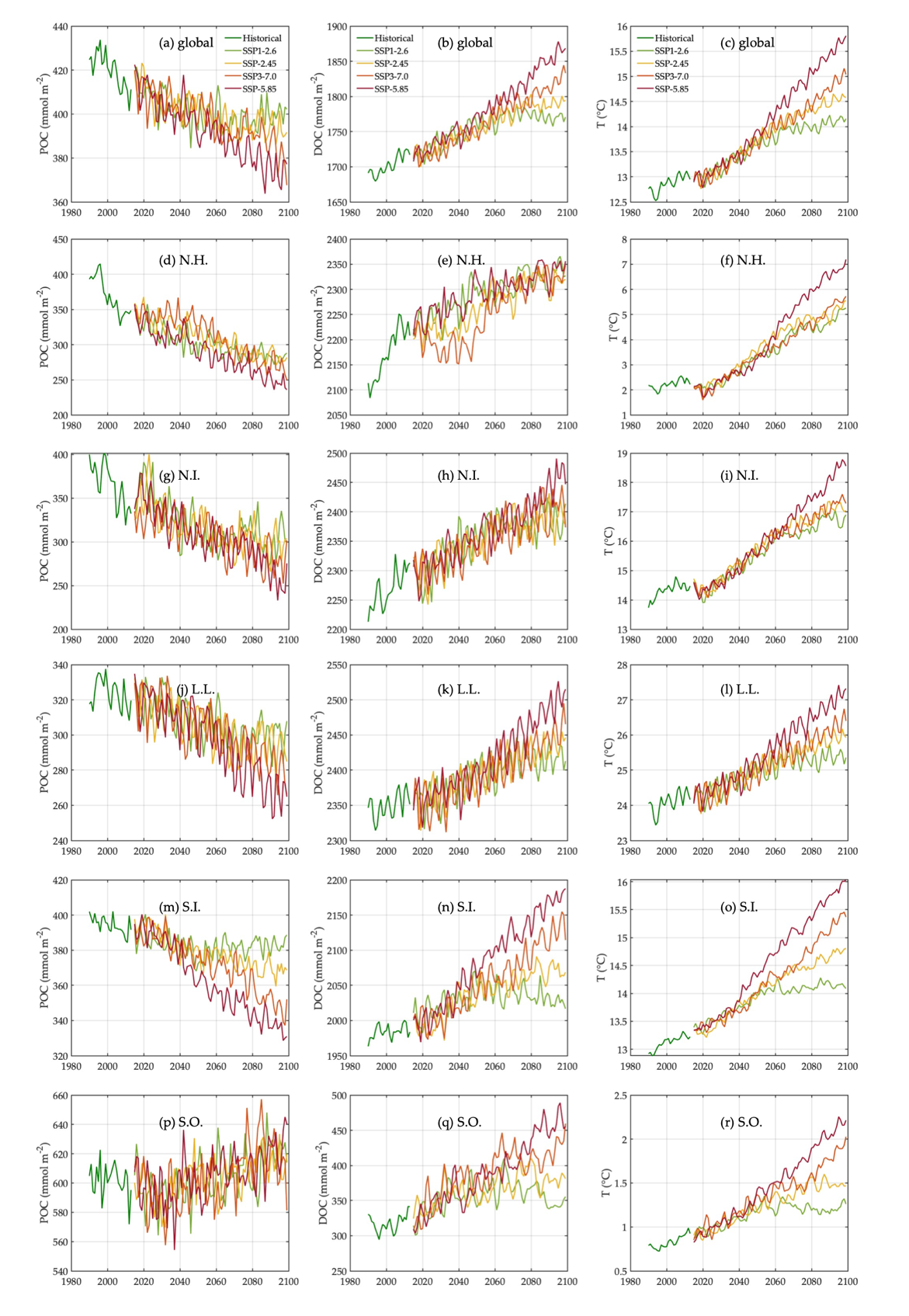
**
